# Personalizing Chinese medicine by integrating molecular features of diseases and herb ingredient information: application to acute myeloid leukemia

**DOI:** 10.18632/oncotarget.16983

**Published:** 2017-04-09

**Authors:** Lin Huang, Haichang Li, Duoli Xie, Tieliu Shi, Chengping Wen

**Affiliations:** ^1^ TCM Clinical Basis Institute, Zhejiang University of Chinese Medicine, Hangzhou, China; ^2^ The Center for Bioinformatics and Computational Biology, Shanghai Key Laboratory of Regulatory Biology, The Institute of Biomedical Sciences and School of Life Sciences, East China Normal University, Shanghai, China

**Keywords:** traditional Chinese medicine (TCM), acute myeloid leukemia(AML), medicine classification, synthetic lethality (SL), drug repurposing

## Abstract

Traditional Chinese Medicine (TCM) has been widely used as a complementary medicine in Acute Myeloid Leukemia (AML) treatment. In this study, we proposed a new classification of Chinese Medicines (CMs) by integrating the latest discoveries in disease molecular mechanisms and traditional medicine theory. We screened out a set of chemical compounds on basis of AML differential expression genes and chemical-protein interactions and then mapped them to Traditional Chinese Medicine Integrated Database. 415 CMs contain those compounds and they were categorized into 8 groups according to the Traditional Chinese Pharmacology. Pathway analysis and synthetic lethality gene pairs were applied to analyze the dissimilarity, generality and intergroup relations of different groups. We defined hub CM pairs and alternative CM groups based on the analysis result and finally proposed a formula to form an effective anti-AML prescription which combined the hub CM pairs with alternative CMs according to patients’ molecular features. Our method of formulating CMs based on patients’ stratification provides novel insights into the new usage of conventional CMs and will promote TCM modernization.

## INTRODUCTION

Acute myeloid leukemia (AML) is one of the hematopoietic malignancies characterized by uncontrolled proliferation of myeloblast. On contrary to advances in cytogenetic analysis at diagnosis and prognostic stratification, the pharmacology therapy researches of AML seem to be barely satisfactory which remain almost unchanged for nearly 40 years [1, 2]. Hematopoietic stem cell transplantation and chemotherapy are the main medical approaches for AML treatment, but the outcome is not ideal with the five year survival rate of AML patients appropriately low to 20% [3]. In addition, only patients under 60 years old are suggested to take chemotherapy because of the incidental grievous damage to normal cells, leaving seldom treatment options for the rest patients [4]. Therefore, it is urgent to propose alternative strategies on AML intervention.

Synthetic lethality (SL) signifies an interaction between two genes which will induce apoptosis when they are simultaneously perturbing the same cells while absence of either gene will take no effect. The increasing number of identified SL gene pairs provides a promising in selective cytotoxicity to tumor cells and meantime reduces side effect of traditional chemotherapy [5]. Therefore, researchers working on AML treatments are seeking the SL therapy. A prototypical example is the application of PARP inhibitors [6] that can effectively kill cancer cells while spare normal tissue [7].

Traditional Chinese Medicine (TCM), especially herbal medicine, as a complementary and alternative medicine treatment, has been widely utilized in cancer treatment. For instance, clinical evidence clarified that therapeutic schedule combined modified Shengma Biejia Decoction with CAG (cytarabine, aclacinomycin and granulocyte colony-stimulating factor) program was beneficial for patients in relieving clinical symptoms and enhancing physical quality [8]. Another ancient drug, *Arsenic* (Pi Shuang), is well known for its substantial therapeutic effect on acute promyelocytic leukemia and has been accepted as a standard treatment [9, 10]. The conventional recognition in Chinese medicines (CMs) is based on various experience of predecessors. However, in the past few decades, scientists have focused their attention on extracting effective chemical components in CMs and unveiling the pharmacological mechanisms of them, contributing to the discovery of anti-cancer herbs such as *Panax notoginseng* (San Qi) [11] and *Pinellia ternate (Ban* Xia) [12]. Although researches on herbal monomer can decipher the therapeutic function of herbs to some extent, they cannot be representative of the complete multi-targets curative effect of herbs and most pharmacological activities in herbs remain underestimated.

Presently, bioinformatics methods are burgeoning in avenues of TCM-related researches like mechanism analysis on complex herbal prescriptions [13]. The emergence of comprehensive databases such as Traditional Chinese Medicine Integrated Database (TCMID) [14] and TCM Database@Taiwan [15] made it possible for large-scale analysis in TCM. Targets of therapeutic compounds can be acquired through various open access databases which remind us of the possibility of delving more specific effect herbs and reclassifying them in a new perspective way.

**Figure 1:**
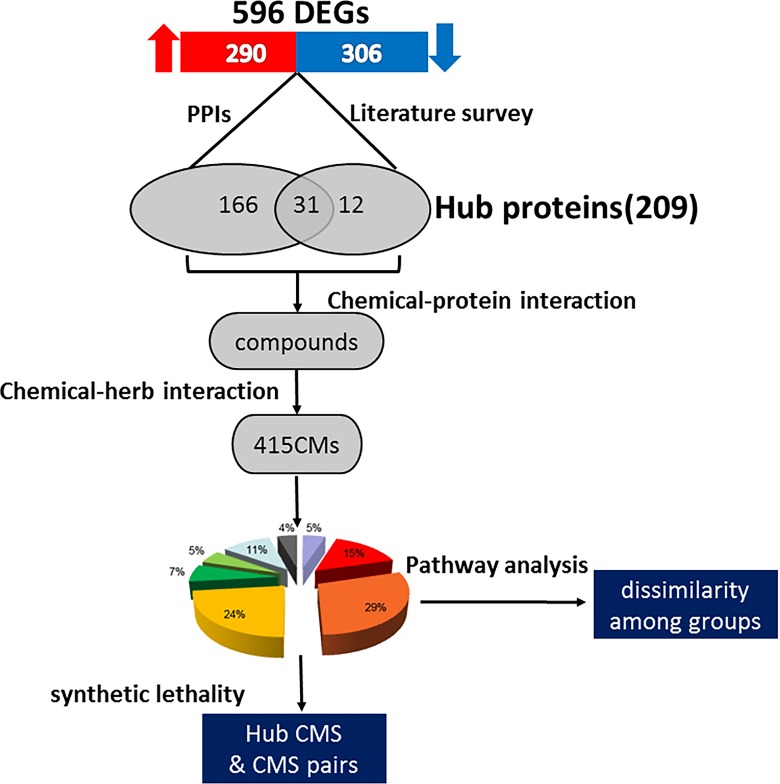
Schematic diagram of the work

In this study, as described in the schematic diagram (Figure 1), we adopted 596 differential expression genes (DEGs) reported based on AML RNA-seq data [16] and detected hub proteins through calculating degree of protein-protein interactions (PPIs) and literature survey. Chemical compounds highly correlated with the hub targets were then acquired. Thereupon, CMs containing those chemical compounds were selected and grouped into 8 categories based on traditional theory of TCM. SL gene pairs and pathway analysis were applied to analyze dissimilarity, generality and intergroup relations of different categories. Finally, we proposed 415 suggested anti-AML CMs and proposed a rational combination scheme to formulate an effective prescription. Our method to select a group of CMs for a special disease by combining the latest research progresses of disease mechanisms with the traditional theory shows promise of modernized personalized CMs and delivers a new prospective on old CMs.

## RESULTS

### Defining hub targets in DEGs

A set of DEGs (596) for AML have been reported based on AML RNA-seq data [16], which includes 306 down-regulated genes and 290 up-regulated genes (Supplementary Table 1). We made a literature survey on the 596 DEGs to identify critical proteins contributing to the progress on AML. The investigation indicated that 43 proteins encoded by DEGs have explicit influence on AML including negative correlation with adverse prognosis (ATP1B1, HOXB4, MN1) [17–19] and promoting cell proliferation (DLL3) [20]. These 43 proteins were defined as key proteins (Supplementary Table 2). Human PPIs were extracted from InWeb_InBioMap database to generate an interaction network [21]. The 596 proteins were mapped to PPI network to identify potential hub proteins with high degree. 149 proteins failed to find any interacted proteins while the rest 447 ones were successfully matched. Totally, 447 proteins have 7,494 interactive partners and the degree of them in the network was listed in descending order in Supplementary Table 3. 197 proteins respectively encoded by 111 down-regulated genes (OBSL1, MYH9, ATXN1, etc.) and 86 up-regulated genes (BMI1, DBN1, CD81, etc.) possess greater than or equal to 25 interactions, covering 31 key proteins. The 197 proteins and the rest 12 key proteins were selected out as hub targeted proteins for the subsequent CMs identification.

### Network-based CMs selection

**Figure 2:**
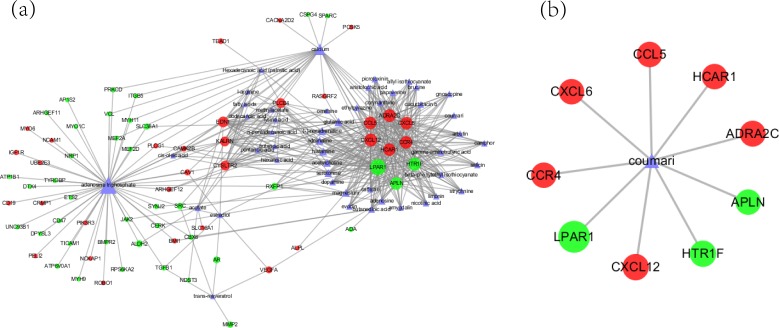
The relationship between hub genes, CM ingredients and herbs **a**. The relationships between the hub genes and ingredients. Layout of node size were adjusted by node degree which was defined by the number of links with other nodes. The green circle stands for down-regulated DEGS; the red circle stands for up-regulated DEGs; the blue triangle stands for CM ingredients. **b**. Relationship between coumari and DEGs.

STITCH compiled reliable experimental and predictive data of chemical-protein interactions along with a detailed score for the confidence interval [22]. Chemical-protein interactions involving one of the 209 selected hub proteins with confidence range greater than or equal to 0.9 (high confidence) were selected which ensure the bioactivity of the compounds. Totally, we obtained 2928 interactions, covering 109 proteins and 963 chemical compounds. To detect the CMs that contain those 963 chemical compounds, we mapped them to TCMID. 122 compounds were successfully matched with CMs’ ingredients in TCMID, covering 592 chemical-protein interactions with 86 DEGs. The chemical compounds included small molecules such as adenosine triphosphate and calcium, and TCM active substance such as coumari and amygdalins (Figure 2a and 2b). Interference on calcium transport through calcium channel blockers has been a successful attempt on delaying the progression of some malignancies including AML which is sensitive to the cytosolic calcium concentration in experiments [23, 24]. Meanwhile, extracellular magnesium concentration might expedite the AML cell differentiation through interfering with calcium homeostasis [25]. However, impact of those TCM active substance on AML has seldom been studied.

458 CMs were selected at the very beginning after mapping the compounds to TCMID. After excluding the unconventional medication, such as *Penis et Testes Callorhini* (Hai Gou Shen), *Penis Cervi* (Lu Bian) and Ferri (Tie pian), and those CMs with severer side effect such as *Aristolochia manshuriensis* (Guan Mu Tong) and *Aristolochia fangchi* (Mu Fang Ji), 415 CMs with complete annotation in TCMID were retained. Top of the list was *Panax ginseng* (Ren Shen) whose ingredients targeted 83 proteins encoded by DEGs (37 up-regulated DEGs and 46 down-related DEGs), followed by the *Cornu Cervi Pantotrichum* (Lu Rong), *Semen Astragali Complanati* (Sha Yuan Zi) and *Cistanche deserticola* (Rou Cong Rong) targeted 71, 61 and 61 proteins respectively.

**Figure 3:**
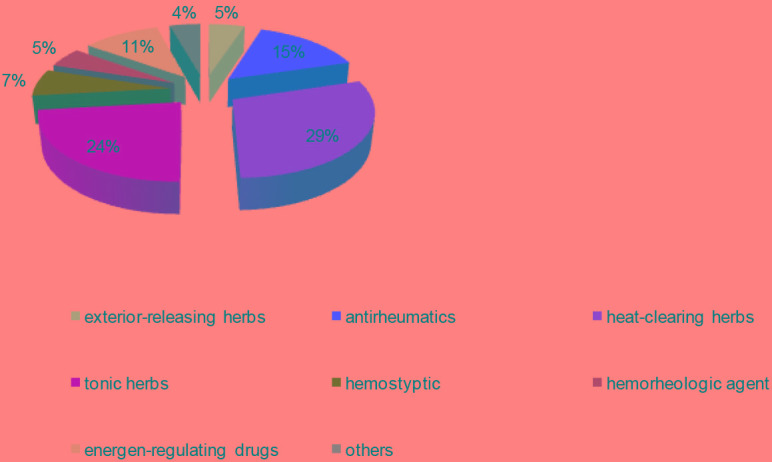
The proportion of CMs in each group This pie chart demonstrates distribution of the 415 in 8 groups.

**Figure 4:**
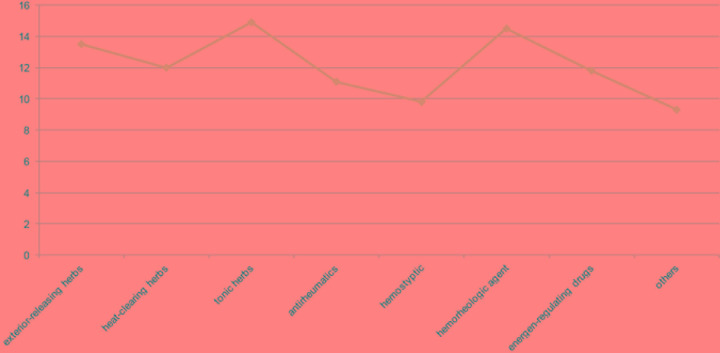
The average number of targeted DEGs in each group The line chart expresses the number of targeted DEGs in each group.

We categorized the 415 CMs into 8 groups in accordance with the Traditional Chinese Pharmacology, namely exterior-releasing herbs (21, 5%), antirheumatics (64, 15%), heat-clearing herbs (123, 29%), tonic herbs (98, 24%), hemostyptic (28, 7%), hemorheologic agent (21, 5%), energen-regulating drugs (45, 11%) and others (18, 18.4%) (Figure 3, Supplementary Table 4). The number of targeted proteins encoded by DEGs was calculated to assess the anti-AML efficiency of groups. The average protein targets for 8 groups were 12.1, among them, tonic herbs, hemorheologic agent and exterior-releasing herbs have the most targets with 14.9, 14.5 and 13.5, respectively (Figure 4). We also found that CMs targeting the most proteins were almost assigned to tonic herbs.

### Pathway analysis

**Figure 5:**
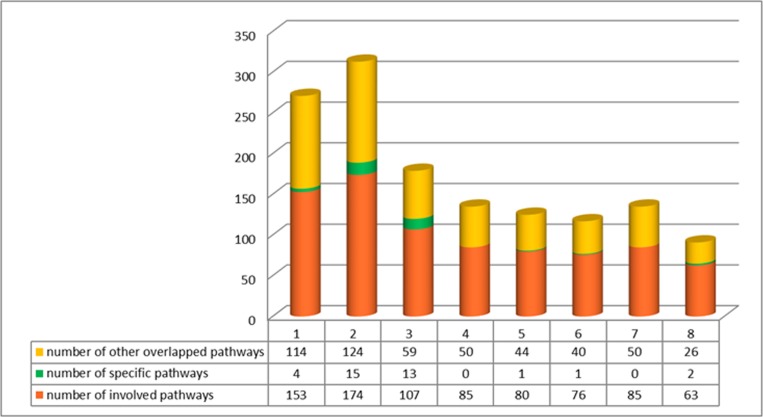
Distribution of KEGG pathways in each group The histogram shows the distribution of KEGG pathways in each group. Number 1 to 8 in the trunnion axis respectively orderly stands for different groups, exterior-releasing herbs, heat-clearing herbs, tonic herbs, antirheumatics, hemostyptic, hemorheologic agent, energen-regulating drugs and others. The orange part stands for the number of total involved KEGG pathways for targets in each group; the green part stands for the unique pathways in each groups; the yellow part stands for the number of pathways that are overlapping with the other 7 groups.

Pathway enrichment analysis was carried out for AML DEGs and targets of each group (Supplementary Table 5, Supplementary Table 6). Enrichment result showed that the AML DEGs were involoved in 23 pathyways while the CM targets were involved in 205 pathways with 17 pathways overlapped between them (Table 1). There were 35 pathways shared among the 8 categories including chemokine signaling pathway, calcium signaling pathway, Fc epsilon RI signaling pathway and neuroactive ligand-receptor interacion, accounting for a 16.8% proportion (Figure 5). The 35 common pathways represented the generality among different groups for targeting the same set of pathways. Chemokine signaling pathway was also one of the AML DEGs involved pathways. Studies comfirmed that chemokine signaling pathway and Fc epsilon RI signaling pathway were sharply activated in HL-60 cells [26]. Activation of intracellular calcium signaling might be a promising way to stimulate leukemia blasting into dendritic cells and make progress on antileukemia vaccine strategies [27].

**Table 1:** Overlapped KEGG pathway among DEGs and 8 categories

KEGG Pathway	Category
Adherens junction	3
Amoebiasis	1,2
Chagas disease (American trypanosomiasis)	1,2
Chemokine signaling pathway	1,2,3,4,5,6,7,8
Cytokine-cytokine receptor interaction	8
Dilated cardiomyopathy	1,2,3,4,5,6,7
Glycerolipid metabolism	3
GnRH signaling pathway	1,2
HIF-1 signaling pathway	2
Hypertrophic cardiomyopathy (HCM)	1,2
Inflammatory mediator regulation of TRP channels	1,2
Pathways in cancer	1,2,3
Proteoglycans in cancer	1,2
Rap1 signaling pathway	1,2
Regulation of actin cytoskeleton	3
Rheumatoid arthritis	2
TNF signaling pathway	1

Except for antirheumatics and energen-regulating drugs, the rest of the categories possessed unique pathways. The common pathways indicated that CMs in different categories played similar function while those unique pathways in each category represented its specific funtions. Tonic herbs seemed to be the most specificity group affecting 12.1% (13 out of 107) peculiar pathways, including acute myeloid leukemia, ribosome and adherens junction. Both ribosome and adherens junction play important role in leukemic stem cell biology and cancer course [28].

### Synthetic lethality gene pairs in CM

Synthetic lethality has been suggested in the cancer treatment, which can selectively initiate apoptosis in cancer cells while spare normal cells. To detect the potential synergetic effect of those ingredients on the targets, we downloaded data of SL gene pairs in homo sapiens from SynLethDB [29]. According to the data, only two gene pairs are related to AML with confidence score > 0.7, i.e. BRCA1-TP53 (0.82046875) and CHEK1-TP53 (0.85). We collected the whole ingredients and targets for those 415 CMs and mapped them to the SL data to investigate whether these ingredients can act on the SL gene pairs. We discovered that ingredients of 5 CMs had effect on high confidence ( > 0.7) AML-related SL gene pairs. Among them, *Cornu Cervi Pantotrichum* (Lu Rong), belonging to the tonic herbs, act on 5 AML-related SL gene pairs (including the 2 high confidence pairs) i.e., BRCA1-TP53 (0.82046875), CHEK1-TP53 (0.85), PNKP-PTPN6 (0.56), ATM-TP53 (0.56) and FLT3-MAPK8 (0.56), while *Panax ginseng* (Ren Shen) works on 4 of them (except for PNKP-PTPN6). The rest three CMs are *Ilex cornuta* (Gou Gu), *Camellia sinensis* (Ye Cha Ye) and *Folium camelliae sinensis* (Lv Cha). In addition to AML-related gene pairs, 4 CMs also can induce other kind of SL which may lead to toxic or side effect. To be detailed, *Cornu Cervi Pantotrichum* (Lu Rong), *Panax ginseng* (Ren Shen), *Camellia sinensis* (Ye Cha Ye) and *Folium camelliae sinensis* (Lv Cha) can induce 65, 66, 3, 3 other SL gene pairs, respectively. These 5 CMs were regarded as effective CMs for AML therapy. Apparently, *Ilex cornuta* (Gou Gu) seems to be the most safety CM. We further looked over the explicit action of the 5 CMs on the 43 key proteins (Supplementary Table 7). The investigation suggested an ambiguous role of Panax ginseng in AML treatment while the other 4 CMs seems to benefit for anti-AML treatment. Although it suppresses cancer invasion through reaction on ITGB5 [30], *Panax ginseng* may also stimulate bone marrow blast by activating CTGF [31]. Hence, we filtered out *Panax ginseng* from the hub CMs. Then, we used medicine data from Drugbank to compare the targets of the 4 CMs with the targets of western medicine using for AML treatment. Until now, only 5 medicines were approved by FDA for AML treatment and a target of *Cornu Cervi Pantotrichum* (Lu Rong), PRKCD, is overlapped with the targets of a recorded medicine Ingenol Mebutate (Accession Number: DB05013) which further verify the effectiveness of the chosen CMs.

**Figure 6:**
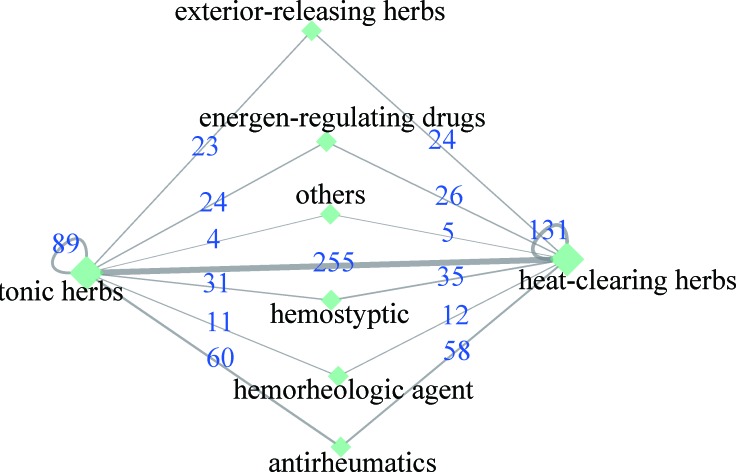
CM pairs formed among different groups The links between groups means that herbs from the two side can induce leukemia-related SL. The width of the links represents the number of herb pairs and the numbers on the links stand for the value of number.

Furthermore, 758 herb-herb pairs (covering 201 kinds of CMs) were suggested to have the function of inducing SL (Supplementary Table 8). As shown in Figure 6, 255 CM pairs formed between tonic herbs and heat-clearing herbs groups, ranking as the highest rates when compared with the rest group combinations. The intra-group effective CM pairs also can only be found in these two groups. When the CMs target the AML-related SL pairs, they also work on other genes with SL effect, which might result in toxic or side effect. Therefore, to avoid the potential severe side effect, it is reasonable to exclude the CMs pairs that affect a high proportion of genes with SL effect that are unrelated to AML in prescription.

### Formulating an effective anti-AML prescription

On basis of above analysis result, we proposed a formula for an effective anti-AML prescription with the combination of hub CMs pairs and alternative CMs according to patients’ molecular features. 4 CMs targeting genes with SL effect were defined as hub CMs in prescription. According to the classification, herbs targeting most proteins encoded by DEGs belong to tonic herbs, hemorheologic agent and exterior-releasing herbs with average value of 14.9, 14.5 and 13.5. Pathway analysis further manifested the vital function of tonic herbs. Fortified SL effect can be acquired through a joint action in hub CMs and the 201 other herbs in which tonic herbs, hemorheologic agent and exterior-releasing herbs are preferable groups to yield the greatest intervention on AML DEGs. The CMs associated for joint action were defined as hub CMs pairs. Varying clinical manifestation or molecular features of a patient will serve as the selective basis of alternative CMs and the 415 CMs would serve as herb set for option. For these reasons, a formula for an effective anti-AML prescription was suggested to be a combination of hub CMs pairs with alternative CMs as following:

Prescription = Hub CMs pairs + Alternatives CMs

## DISCUSSION

TCM, as a traditional medical intervention, has been widely used in Asian countries for several thousand years. One of the characteristics and advantages in TCM is individualized treatment which is coincident with the mission of current popular precision medicine. Recognition approaches for CMs have altered from semplice experience accumulation to experimental researches aiming at compound identification. However, in clinical application, physicians still face the challenge in research-based CMs chosen because of the complexity of herbal compounds. Hence, we try to meliorate conventional TCM practice with modern medical technology by integrating molecular features of diseases and CMs ingredient information.

AML DEGs, human PPIs and herbal compounds were jointly exploited to select the potential effective CMs for AML treatment. Finally, we screened 415 from 8,159 compiled CMs in TCMID. Some of the herbs have been proved to be beneficial for leukemia therapy. For example, *Semen Astragali Complanati* (Sha Yuan Zi) was found to inhibit acute promyelocytic leukemia cell line HL-60 [32]. In addition, proliferation of leukemia cell line K562 was likely to be suppressed by echinacoside, one ingredient of *Cistanche deserticola* (Rou Cong Rong) [33]. The major herbs of Shengma Bie-jia Decoction (*Cimicifuga dahurica* [Sheng Ma], *Carapax Trionycis* [Bie Jia], *Angelica sinensis* [Dang Gui] and *Glycyrrhiza uralensis* [Gan Cao]) has been verified to be beneficial for relieving clinical symptoms, are also in our screened CMs.

**Figure 7:**
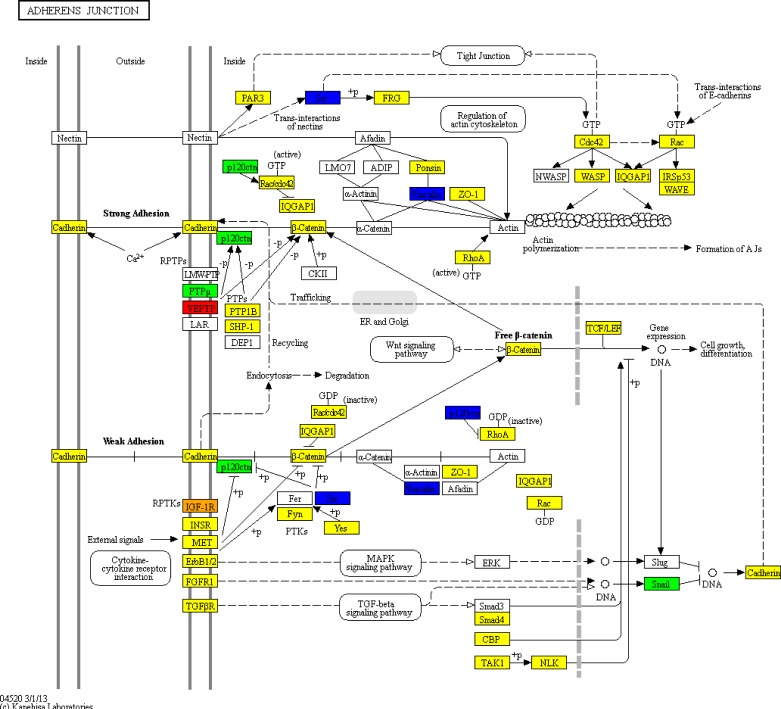
Adherens junction pathway in KEGG The red nodes stand for up-regulated DEGs; the green nodes stand for down-regulated DEGs; the blue nodes stand for the overlapped genes in down-regulated DEGs and target of tonic herbs; the orange node stands for the overlapped genes in up-regulated DEGs and target of tonic herbs; the yellow nodes stand for targets of tonic herbs.

According to the classification, herbs targeting most proteins encoded by DEGs belong to tonic herbs, hemorheologic agent and exterior-releasing herbs. Pathway analysis for the targets of 8 CMs groups revealed the function generality and peculiarity in each group. Chemokine signaling pathway is the only one involved in each of the 8 groups and AML DEGs, indicating a common biological process among groups. The enriched specific pathways in each group are responsible for the function peculiarity. Adherens junction is also one of the pathway influenced by AML DEGs. As shown in Figure 7, targets of the group almost filled up proteins between the up-regulated DEGs and down-regulated DEGs in the pathway.

SL is a double-edged sword in cancer treatment. Based on the data in SynLethDB, we identified 4 herbs whose ingredients could target high confidence AML-related SL gene pairs, so as 758 herb-herb pairs coming from 201 herbs, which suggested 201 (48.3%) screened CMs might be potential drugs for selective AML treatment (Table 2, Supplementary Table 8). Each herb-herb pair contains at least one of the hub CMs. In consideration of drug safety, CM pairs with high proportion of AML-related SL gene pairs and targeting few other SL pairs are recommended in clinical practice.

**Table 2:** List of CMs and the involving SL gene pairs and ingredients

CM name	Ingredient 1	Target 1	Ingredient 2	Target 2	confidence scores
Arachis hypogaea	resveratrol	ATM	resveratrol	TP53	0.75
Camellia sinensis	caffeine	ATM	caffeine	TP53	0.75
Camellia sinensis	caffeine	ATM	epigallocatechin 3-gallate	TP53	0.75
Camellia sinensis	caffeine	CHEK1	caffeine	TP53	0.85
Camellia sinensis	caffeine	CHEK1	epigallocatechin 3-gallate	TP53	0.85
Capsicum annuum	capsaicin	ATM	capsaicin	TP53	0.75
Cornu Cervi Pantotrichum	adenosine triphosphate	BRCA1	adenosine triphosphate	TP53	0.82
Cornu Cervi Pantotrichum	adenosine triphosphate	ATM	adenosine triphosphate	TP53	0.75
Cornu Cervi Pantotrichum	adenosine triphosphate	CHEK1	adenosine triphosphate	TP53	0.85
Cistanche deserticola	genistein	ATM	lysine acid	TP53	0.75
Cistanche deserticola	genistein	ATM	lysine	TP53	0.75
Cistanche deserticola	genistein	ATM	zinc	TP53	0.75
Diospyros kaki	genistein	ATM	quercetin	TP53	0.75
Folium camelliae sinensis	caffeine	ATM	caffeine	TP53	0.75
Folium camelliae sinensis	caffeine	CHEK1	caffeine	TP53	0.85
fructus Sophorae	genistein	ATM	quercetin	TP53	0.75
Gardenia jasminoides	genistein	ATM	zinc	TP53	0.75
Gardenia jasminoides	genistein	ATM	quercetin	TP53	0.75
Glehnia littoralis	Vanilloid	ATM	Vanilloid	TP53	0.75
Glehnia littoralis	Vanilloid	ATM	quercetin	TP53	0.75
Ilex cornuta	caffeine	ATM	caffeine	TP53	0.75
Ilex cornuta	caffeine	CHEK1	caffeine	TP53	0.85
Imperata cylindrica var. major	Vanilloid	ATM	Vanilloid	TP53	0.75
Morus alba	resveratrol	ATM	resveratrol	TP53	0.75
Panax ginseng	adenosine triphosphate	BRCA1	adenosine triphosphate	TP53	0.82
Panax ginseng	adenosine triphosphate	ATM	adenosine triphosphate	TP53	0.75
Panax ginseng	adenosine triphosphate	CHEK1	adenosine triphosphate	TP53	0.85
Polygonum cuspidatum	trans-resveratrol	ATM	trans-resveratrol	TP53	0.75
Polygonum cuspidatum	trans-resveratrol	ATM	resveratrol	TP53	0.75
Polygonum cuspidatum	trans-resveratrol	ATM	quercetin	TP53	0.75
Polygonum cuspidatum	resveratrol	ATM	trans-resveratrol	TP53	0.75
Polygonum cuspidatum	resveratrol	ATM	resveratrol	TP53	0.75
Polygonum cuspidatum	resveratrol	ATM	quercetin	TP53	0.75
Polygonum multiflorum	resveratrol	ATM	resveratrol	TP53	0.75
Radix Cudraniae	resveratrol	ATM	resveratrol	TP53	0.75
Rheum wittrocki	resveratrol	ATM	resveratrol	TP53	0.75
Semen Phaseoli	resveratrol	ATM	resveratrol	TP53	0.75
Semen Sojae Praeparata	genistein	ATM	arsenicum	TP53	0.75
Semen Sojae Praeparata	genistein	ATM	lysine	TP53	0.75
Smilax glabra	trans-resveratrol	ATM	trans-resveratrol	TP53	0.75
Smilax glabra	trans-resveratrol	ATM	resveratrol	TP53	0.75
Smilax glabra	resveratrol	ATM	trans-resveratrol	TP53	0.75
Smilax glabra	resveratrol	ATM	resveratrol	TP53	0.75
Smilax menispermoidea	resveratrol	ATM	resveratrol	TP53	0.75
Sophora japonica	genistein	ATM	quercetin	TP53	0.75
Testa Arachidis Hypogaeae	resveratrol	ATM	resveratrol	TP53	0.75
Testa Arachidis Hypogaeae	resveratrol	ATM	quercetin	TP53	0.75
Thalictrum ichangense	capsaicin	ATM	capsaicin	TP53	0.75
Trifolium pratense	genistein	ATM	quercetin	TP53	0.75
Vatica rassak	resveratrol	ATM	resveratrol	TP53	0.75
Veratrum album	resveratrol	ATM	resveratrol	TP53	0.75
Veratrum grandiflorum	resveratrol	ATM	resveratrol	TP53	0.75
Veratrum nigrum var. ussuriense	resveratrol	ATM	resveratrol	TP53	0.75
Vitis vinifera	resveratrol	ATM	resveratrol	TP53	0.75

In TCM theory, heat-clearing herbs, exterior-releasing herbs and hemorheologic agent are mostly applied in the early stage of cancers while tonic herbs are widely used in the late stages of cancer when patients have weakened immune system resulting from cancer treatment such as chemotherapy. Whereas, our work found out that an appropriate combination between tonic herbs and other CMs such as heat-clearing herbs and hemorheologic agent might be beneficial for AML patients in the entire disease treatment process. CMs pairs in Table 2 which contains at least one of the hub CMs is offered for choosing the hub CMs combination. Variations of clinical manifestation or molecular features of a patient will serve as the selective basis of alternative CMs.

To demonstrate the application of our strategy for personalized CMs, we took Shengma Biejia Decoction as an example. We found that the major herbs of Shengma Biejia Decoction belong to tonic herbs (Glycyrrhiza uralensis [Gan Cao], Carapax Trionycis [Bie Jia] and Angelica sinensis [Dang Gui]), the most essential CM group, while Cimicifuga dahurica (Sheng Ma) is one of the exterior-releasing herbs which serve as fever reducer. To make the formula comply with AML patients with different molecular features, we did some modification to it. As indicated in the analysis result, 3 hub CMs, *Camellia sinensis* (Ye Cha Ye), *Folium camelliae sinensis* (Lv Cha) and *Ilex cornuta*(Gou Gu), can induce SL when combined with *Angelica sinensis* (Dang Gui). To maximize the targeting proteins encoded by DEGs in the formula, we proposed to add *Folium camelliae sinensis* (Lv Cha) (or *Camellia sinensis* [Ye Cha Ye]), one of heat-clearing herbs as the extension, because 3 CMs in the decoction are already from tonic herbs.

**Figure 8:**
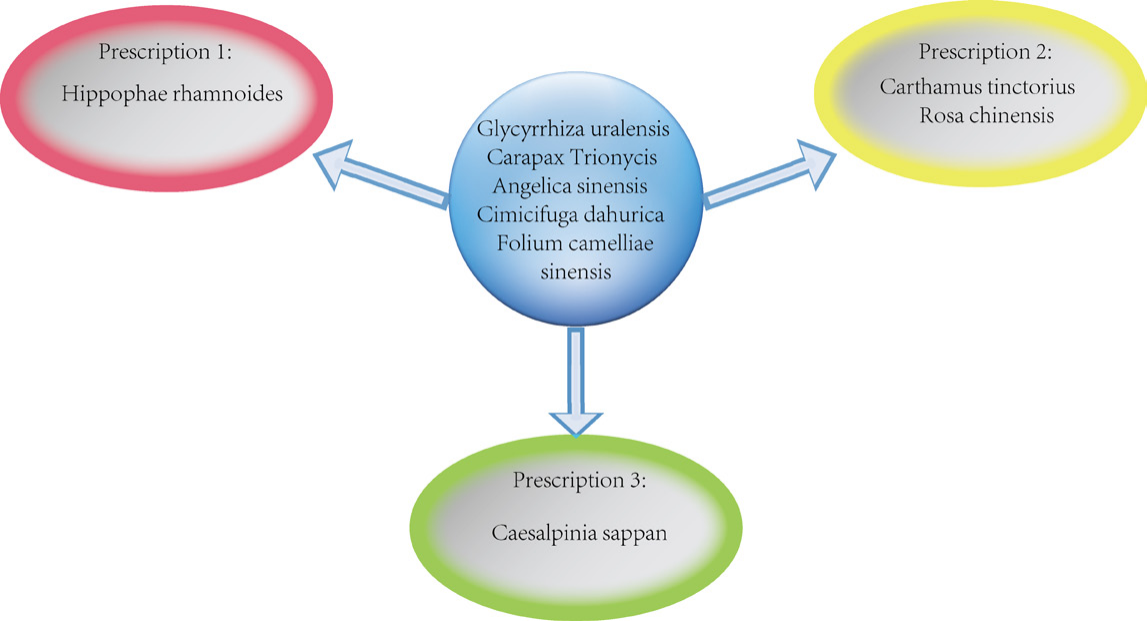
Three prescription derived from of Shengma-Biejia Decoction The figure shows three prescription derived from Shengma-Biejia Decoction.

To further enhance the SL effect with more coverage of the targeting proteins, we selected CMs from hemorheologic agent group based on the 758 identified herb-herb pairs, resulting in 4 frequently-used herbs. Based on the effect variation of the 4 herbs, Shengma-Biejia Decoction can be formulated into 3 different prescriptions for 3 different clinical features (Figure 8). To be specific, prescription 1 is appropriate for patients suffering expectoration and prescription 2 is for patients with significant abdominal pain syndrome, while prescription 3 is appropriate for patients suffering joint pain.

Currently, no systematic clinical evidence has proved that TCM can significantly influence the survival rates of any cancers, but as a complementary therapy, TCM has shown its value [34]. Nevertheless, during the past hundreds of years, guiding theory of the usage of CM remains nearly unchanged and progresses in latest achievements of herbs have not been incorporated into the practice of TCM, which seriously restrict the development of TCM. The wide usage of next generation technologies in the biomedical fields has greatly facilitated our understanding of underlying mechanisms of various diseases, including cancers, which promotes the disease treatments into precision medicine era. Considering that individual difference on molecular level can be detected by regular test in the nearly future, personalized TCM treatment would be synchronously realized. Our method of formulating CMs based on patient underlying molecular features provides novel insights into the new usage of conventional CMs and will promote TCM modernization.

## MATERIALS AND METHODS

### Different expression genes in AML patients

We adopted the DEGs in AML patients identified by Zachary J Faber and his colleagues in 2016. A total of 596 DEGs were figured out in their research by carrying out whole-genome or whole-exome sequencing analysis for 165 samples (both adults and pediatric) with RUNX1-RUNX1T1 or CBFB-MYH11 rearrangements.

### Protein-protein interaction data

Homo sapiens protein-protein interaction data was extracted from InWeb_InBioMap, Proteins encoded by DEGs were mapped to the global PPIs to seek out the hub proteins. In this step, we identified 197 hub proteins which harbored greater than or equal to 25 interactions as the hub proteins for herb selection. Some studies defined the hub proteins as those ones proteins that who interacted with ≥10 partners [35]. The criterion was set over 10 years ago by that time, the human protein interactions have not been recognized so comprehensive. In our study, for the chosen DEGs, more than half of them interacted with ≥ 10 partners based on currently enriched protein interaction data, it indicates that the previous criterion for hub protein selection is no longer applicable in current study. Thus, we took a 1.5-fold change to the suggested number (≥25) which covered only 20% of the DEGs. The networks visualization was realized through Cytoscape software (version 3.4.0) [36].

### Chemical data collection

Search Tool for Interacting Chemicals Databases (STITCH 5, http://stitch.embl.de) provides interactions between proteins and small molecules. Each interaction was along with a predicted confidence score which 0.7 was suggested as the divider line for high confidence [37]. We raised the cutoff of confidence score for chemical chosen to 0.9 to ensure the biological activities. After the filtration, 961 chemical compounds correlating with 108 hub proteins were picked out.

### Chinese medicine mapping

Traditional Chinese Medicine Integrated Database (TCMID, http://www.megabionet.org/tcmid/) is one of the authority available database for recording Chinese herb ingredients and herbs. The above-mentioned 961 chemical compounds were mapped to TCMID for herb selection and 106 compounds were successfully matched with ingredients of herbs. Finally, 415 herbs with complete annotation were singled out. Those 415 herbs were them classified into 8 categories according to the Traditional Chinese Pharmacology.

### Synthetic lethality gene pairs

Synthetic lethality database (SynLethDB, http://histone.sce.ntu.edu.sg/SynLethDB/) complied SL gene pairs on human species. Since the SL gene pairs may cause side effects, we lowered the scores to 0.7 instead of 0.9 as the cutoff for SL gene pairs chosen. Then the mapping result found 201 CMs involved in the AML-related SL processes.

### Pathway enrichment analysis

Pathway analysis were performed through the utilization of DAVID Bioinformatics Resources 6.8 (DAVID, http://david.ncifcrf.gov/) [38]. Both DEGs and herbal targets of the 8 groups were put into pathway analysis. Pathway visualization was realized through online tool of KEGG.

## SUPPLEMENTARY MATERIALS AND TABLES





## Abbreviations

TCM: Traditional Chinese Medicine
AML: Acute Myeloid Leukemia
CM: Chinese medicine
DEG: differential expression gene
TCMID: Traditional Chinese Medicine Integrated Database
SL: synthetic lethality
CAG: cytarabine, aclacinomycin and granulocyte colony-stimulating facto
PPI: protein-protein interaction
OBSL1: obscurin like 1
MYH9: myosin heavy chain 9
ATXN1: ataxin 1
BMI1: BMI1 proto-oncogene, polycomb ring finger
DBN1: drebrin 1
CD81: CD81 molecule
BRCA1: BRCA1, DNA repair associated
ATM: ATM serine/threonine kinase
CHEK1: checkpoint kinase 1
TP53: tumor protein p53
RUNX1: runt related transcription factor 1
RUNX1T1: RUNX1 translocation partner 1
PRKCD: protein kinase C delta
CBFB: core-binding factor beta subunit
MYH11: myosin heavy chain 11
STITCH: Search Tool for Interacting Chemicals Databases
SynLethDB: Synthetic lethality database.
