# Supplementary material for: Personalizing Chinese medicine by integrating molecular features of diseases and herb ingredient information: application to acute myeloid leukemia
Source: Oncotarget. 2017 Apr 9;8(26):43579–91. doi: 10.18632/oncotarget.16983 (PMC5522171; doi:10.18632/oncotarget.16983)
Supplement: Supplementary file 1 [file oncotarget-08-43579-s001.pdf]

Personalizing Chinese medicine by integrating molecular features of diseases and herb ingredient information: application to acute myeloid leukemia

Supplementary Material

For Supplementray Tables 1,2,3,4,5,6,7,8 see in Supplementary Files
