# Supplementary material for: Personalizing Chinese medicine by integrating molecular features of diseases and herb ingredient information: application to acute myeloid leukemia
Source: Oncotarget. 2017 Apr 9;8(26):43579–91. doi: 10.18632/oncotarget.16983 (PMC5522171; doi:10.18632/oncotarget.16983)
Supplement: Supplementary file 2 [file oncotarget-08-43579-s002.docx]

**Table 1**. 596 differential differential expression genes.

A list of 596 differential expression genes identified by Zachary J Faber and his colleagues.

| **Gene Name** | **Regulation type** | **Gene Name** | **Regulation type** |
| --- | --- | --- | --- |
| MYH11 | Down-regulated | ANXA11 | Down-regulated |
| MEIS1-AS2 | Down-regulated | TNFAIP2 | Down-regulated |
| MEGF10 | Down-regulated | ARF3 | Down-regulated |
| CLIP3 | Down-regulated | HOXB-AS2 | Down-regulated |
| TNFRSF11A | Down-regulated | BEX4 | Down-regulated |
| EMILIN1 | Down-regulated | RP11-103J8.1 | Down-regulated |
| HOXB3 | Down-regulated | HS3ST3A1 | Down-regulated |
| RP11-346D6.6 | Down-regulated | IRF5 | Down-regulated |
| CCL1 | Down-regulated | RUNX1T1 | Up-regulated |
| APLN | Down-regulated | EVC2 | Up-regulated |
| HOXB4 | Down-regulated | PODXL2 | Up-regulated |
| RP1-278O22.2 | Down-regulated | EVC | Up-regulated |
| MMP14 | Down-regulated | CRMP1 | Up-regulated |
| SULF2 | Down-regulated | SIPA1L2 | Up-regulated |
| AK5 | Down-regulated | GS1-5L10.1 | Up-regulated |
| RHBDL3 | Down-regulated | IGSF1 | Up-regulated |
| CLDN12 | Down-regulated | DOCK6 | Up-regulated |
| RP11-640A1.3 | Down-regulated | EIF5A2 | Up-regulated |
| RHBDF1 | Down-regulated | BCL9 | Up-regulated |
| HOXB2 | Down-regulated | PLCG1 | Up-regulated |
| ARHGAP21 | Down-regulated | KIF25-AS1 | Up-regulated |
| IL17RA | Down-regulated | NR6A1 | Up-regulated |
| RERE | Down-regulated | RP11-52L5.6 | Up-regulated |
| TANC1 | Down-regulated | JMY | Up-regulated |
| SLC4A3 | Down-regulated | CPNE7 | Up-regulated |
| CECR6 | Down-regulated | MLLT4 | Up-regulated |
| EXOC3L2 | Down-regulated | HDX | Up-regulated |
| JAG1 | Down-regulated | C2orf54 | Up-regulated |
| RFX8 | Down-regulated | RP11-13K12.1 | Up-regulated |
| CRIM1 | Down-regulated | RP3-470B24.5 | Up-regulated |
| NDST3 | Down-regulated | AC005932.1 | Up-regulated |
| FAM101B | Down-regulated | RAB3IP | Up-regulated |
| SNAI1 | Down-regulated | PTPRN2 | Up-regulated |
| SHD | Down-regulated | GPR144 | Up-regulated |
| CTD-3179P9.1 | Down-regulated | POU4F1 | Up-regulated |
| CTD-2275D24.4 | Down-regulated | IL5RA | Up-regulated |
| MUSK | Down-regulated | RP11-366L20.2 | Up-regulated |
| AL122127.25 | Down-regulated | BMP3 | Up-regulated |
| CAPG | Down-regulated | C6orf132 | Up-regulated |
| RP11-556E13.1 | Down-regulated | ARID5B | Up-regulated |
| PRKCD | Down-regulated | TEAD1 | Up-regulated |
| SLC43A3 | Down-regulated | DNAH8 | Up-regulated |
| EFHC2 | Down-regulated | SBK2 | Up-regulated |
| MPG | Down-regulated | C20orf112 | Up-regulated |
| COBLL1 | Down-regulated | RP11-798K23.1 | Up-regulated |
| RP11-610P16.1 | Down-regulated | CACNA2D2 | Up-regulated |
| PPM1J | Down-regulated | B9D1 | Up-regulated |
| MEF2A | Down-regulated | CTB-26E19.1 | Up-regulated |
| PIK3AP1 | Down-regulated | LINC00958 | Up-regulated |
| ARHGEF11 | Down-regulated | FAM84B | Up-regulated |
| RP1-278O22.1 | Down-regulated | AC141928.1 | Up-regulated |
| LCP1 | Down-regulated | TANC2 | Up-regulated |
| PACSIN1 | Down-regulated | RGS10 | Up-regulated |
| STARD13 | Down-regulated | MYRF | Up-regulated |
| MAFK | Down-regulated | COL23A1 | Up-regulated |
| ITGB7 | Down-regulated | CAMK2B | Up-regulated |
| DENND6B | Down-regulated | HCAR1 | Up-regulated |
| MN1 | Down-regulated | DBN1 | Up-regulated |
| TMEM184A | Down-regulated | RASAL1 | Up-regulated |
| DPYSL3 | Down-regulated | CEACAM19 | Up-regulated |
| CPVL | Down-regulated | SFMBT1 | Up-regulated |
| SASH3 | Down-regulated | RP11-588H23.3 | Up-regulated |
| CDC42BPB | Down-regulated | MGLL | Up-regulated |
| CUEDC1 | Down-regulated | CACHD1 | Up-regulated |
| XPNPEP2 | Down-regulated | DDIT4L | Up-regulated |
| AGPAT9 | Down-regulated | WIPF3 | Up-regulated |
| KIAA0125 | Down-regulated | ABTB1 | Up-regulated |
| RPH3AL | Down-regulated | ERVFRD-1 | Up-regulated |
| RP11-256I23.2 | Down-regulated | RP11-13K12.5 | Up-regulated |
| RP11-451B8.1 | Down-regulated | SLC7A10 | Up-regulated |
| FGD6 | Down-regulated | CDC42BPG | Up-regulated |
| RP11-343K8.3 | Down-regulated | SELE | Up-regulated |
| CSPG4 | Down-regulated | SAMD14 | Up-regulated |
| AF064858.8 | Down-regulated | COL27A1 | Up-regulated |
| IQSEC2 | Down-regulated | LHX6 | Up-regulated |
| CLNK | Down-regulated | PGPEP1L | Up-regulated |
| PLD3 | Down-regulated | ACKR2 | Up-regulated |
| RP11-830F9.6 | Down-regulated | LINC00954 | Up-regulated |
| HPCAL1 | Down-regulated | CAV1 | Up-regulated |
| NRP1 | Down-regulated | PALM | Up-regulated |
| SYNJ2 | Down-regulated | ZNF358 | Up-regulated |
| MEIS1 | Down-regulated | RBFOX3 | Up-regulated |
| BIK | Down-regulated | CD226 | Up-regulated |
| GPR183 | Down-regulated | U82695.10 | Up-regulated |
| IQCD | Down-regulated | DLL3 | Up-regulated |
| CTC-782O7.1 | Down-regulated | RNF112 | Up-regulated |
| WWC2 | Down-regulated | VEGFA | Up-regulated |
| SERPINE1 | Down-regulated | LA16c-329F2.1 | Up-regulated |
| RP11-497G19.3 | Down-regulated | CD81 | Up-regulated |
| PLXNB2 | Down-regulated | ROBO1 | Up-regulated |
| SUSD1 | Down-regulated | DIRC3 | Up-regulated |
| RPS6KA2 | Down-regulated | KALRN | Up-regulated |
| CD47 | Down-regulated | ANXA3 | Up-regulated |
| PLP2 | Down-regulated | ITGB4 | Up-regulated |
| DSE | Down-regulated | RP11-616M22.3 | Up-regulated |
| RAP1GAP2 | Down-regulated | CTC-510F12.2 | Up-regulated |
| CDK2AP1 | Down-regulated | SMPDL3B | Up-regulated |
| AP1S2 | Down-regulated | PTX4 | Up-regulated |
| GSR | Down-regulated | ARHGEF12 | Up-regulated |
| FAM171A1 | Down-regulated | PLEKHA8 | Up-regulated |
| MSR1 | Down-regulated | RP11-309I15.1 | Up-regulated |
| TMEM150B | Down-regulated | GJA4 | Up-regulated |
| MANSC1 | Down-regulated | COL14A1 | Up-regulated |
| MMP2 | Down-regulated | FAM213A | Up-regulated |
| ADA | Down-regulated | ZFP92 | Up-regulated |
| TNS3 | Down-regulated | RP11-460I13.2 | Up-regulated |
| ATP1B1 | Down-regulated | RANBP17 | Up-regulated |
| CRYBG3 | Down-regulated | CHST3 | Up-regulated |
| KRT8 | Down-regulated | LAX1 | Up-regulated |
| VOPP1 | Down-regulated | VLDLR | Up-regulated |
| PLBD1 | Down-regulated | COL6A2 | Up-regulated |
| DFNA5 | Down-regulated | RTKN2 | Up-regulated |
| CD1B | Down-regulated | EFCAB8 | Up-regulated |
| RP11-443G13.2 | Down-regulated | SLC2A7 | Up-regulated |
| ITGB5 | Down-regulated | PCNXL2 | Up-regulated |
| SLC1A4 | Down-regulated | LRP3 | Up-regulated |
| ATP6V0A1 | Down-regulated | KCNMA1 | Up-regulated |
| LAT2 | Down-regulated | C20orf203 | Up-regulated |
| TMEM25 | Down-regulated | RP11-328M4.2 | Up-regulated |
| LPAR4 | Down-regulated | TSPAN15 | Up-regulated |
| LILRA4 | Down-regulated | ELOVL7 | Up-regulated |
| ORAI1 | Down-regulated | BAI2 | Up-regulated |
| AC005162.4 | Down-regulated | MBOAT2 | Up-regulated |
| LINC00173 | Down-regulated | NT5DC3 | Up-regulated |
| CTNND1 | Down-regulated | ACP6 | Up-regulated |
| BMPR2 | Down-regulated | TRIM32 | Up-regulated |
| MIF4GD | Down-regulated | SLC5A10 | Up-regulated |
| KCNK16 | Down-regulated | HIP1R | Up-regulated |
| EMR2 | Down-regulated | RP11-930O11.1 | Up-regulated |
| LAMB3 | Down-regulated | BMP5 | Up-regulated |
| SPNS3 | Down-regulated | IGSF10 | Up-regulated |
| TMEM163 | Down-regulated | BIRC7 | Up-regulated |
| SERPINF1 | Down-regulated | NCKAP1 | Up-regulated |
| CYP2E1 | Down-regulated | ZNF662 | Up-regulated |
| FAM225A | Down-regulated | PLXNB3 | Up-regulated |
| LRRC36 | Down-regulated | LPIN3 | Up-regulated |
| RP11-731D1.4 | Down-regulated | MYO6 | Up-regulated |
| RP11-768G7.2 | Down-regulated | FOXP4 | Up-regulated |
| RP11-73G16.2 | Down-regulated | 7SK | Up-regulated |
| NKD2 | Down-regulated | CPE | Up-regulated |
| FLNC | Down-regulated | WDR35 | Up-regulated |
| RP11-705C15.3 | Down-regulated | FOXL1 | Up-regulated |
| GSTM2 | Down-regulated | CDK14 | Up-regulated |
| RP11-760N9.1 | Down-regulated | CTGF | Up-regulated |
| PRTFDC1 | Down-regulated | FBXO32 | Up-regulated |
| GLIPR2 | Down-regulated | RP11-180P8.3 | Up-regulated |
| SLC38A1 | Down-regulated | MIAT | Up-regulated |
| RP11-165A20.3 | Down-regulated | SERPINE2 | Up-regulated |
| SPINK2 | Down-regulated | PTPRB | Up-regulated |
| PTPRM | Down-regulated | EGFL6 | Up-regulated |
| RP11-432B6.3 | Down-regulated | PIK3R3 | Up-regulated |
| DTX4 | Down-regulated | C20orf195 | Up-regulated |
| C15orf38 | Down-regulated | ACSM1 | Up-regulated |
| PCCA | Down-regulated | AC093110.3 | Up-regulated |
| ATXN1 | Down-regulated | EBI3 | Up-regulated |
| TNFSF13 | Down-regulated | PRAME | Up-regulated |
| ITM2A | Down-regulated | SRPX | Up-regulated |
| TRERNA1 | Down-regulated | SLC22A31 | Up-regulated |
| AF064858.11 | Down-regulated | PLEKHB1 | Up-regulated |
| RP11-384F7.2 | Down-regulated | BAG2 | Up-regulated |
| RD3L | Down-regulated | MCF2L | Up-regulated |
| RP11-705C15.5 | Down-regulated | NR5A1 | Up-regulated |
| MAGIX | Down-regulated | PAPPA | Up-regulated |
| FAM225B | Down-regulated | C2orf66 | Up-regulated |
| CBFA2T3 | Down-regulated | GPM6B | Up-regulated |
| TLR7 | Down-regulated | ACSM3 | Up-regulated |
| SNX30 | Down-regulated | EMP2 | Up-regulated |
| MEIS1-AS1 | Down-regulated | PELI2 | Up-regulated |
| WDFY4 | Down-regulated | ZHX2 | Up-regulated |
| RP11-856M7.4 | Down-regulated | GLCCI1 | Up-regulated |
| PLXDC2 | Down-regulated | FAM115C | Up-regulated |
| SCHIP1 | Down-regulated | RP11-353N4.5 | Up-regulated |
| OBSL1 | Down-regulated | NOV | Up-regulated |
| RP11-390P2.4 | Down-regulated | RP11-403I13.9 | Up-regulated |
| MYO1C | Down-regulated | ASIC1 | Up-regulated |
| IDH3A | Down-regulated | ENPP4 | Up-regulated |
| RP11-678G15.2 | Down-regulated | RP5-884M6.1 | Up-regulated |
| SPARC | Down-regulated | CCL18 | Up-regulated |
| STAB1 | Down-regulated | MIR145 | Up-regulated |
| ILDR2 | Down-regulated | SLAIN1 | Up-regulated |
| ELF4 | Down-regulated | CROCC | Up-regulated |
| KRT18 | Down-regulated | KIF21A | Up-regulated |
| BTG2 | Down-regulated | RASSF8 | Up-regulated |
| PQLC2 | Down-regulated | CD58 | Up-regulated |
| MYH9 | Down-regulated | RAB3C | Up-regulated |
| IL3RA | Down-regulated | RP11-137H2.6 | Up-regulated |
| CD9 | Down-regulated | RNF215 | Up-regulated |
| TOP1MT | Down-regulated | ADRM1 | Up-regulated |
| C20orf166 | Down-regulated | NRBP2 | Up-regulated |
| ANO7 | Down-regulated | PARD3B | Up-regulated |
| CD1E | Down-regulated | TCN1 | Up-regulated |
| TM4SF19-AS1 | Down-regulated | EMID1 | Up-regulated |
| RP11-400F19.18 | Down-regulated | ADRA2C | Up-regulated |
| CCDC175 | Down-regulated | MYO18B | Up-regulated |
| RP11-431I8.1 | Down-regulated | RP11-141M3.5 | Up-regulated |
| LCT | Down-regulated | TMEM44 | Up-regulated |
| SH3BP2 | Down-regulated | AP001468.58 | Up-regulated |
| P2RX3 | Down-regulated | BMI1 | Up-regulated |
| SGSH | Down-regulated | SLC16A1 | Up-regulated |
| SCPEP1 | Down-regulated | DOK6 | Up-regulated |
| ANPEP | Down-regulated | ARHGEF25 | Up-regulated |
| AIM1 | Down-regulated | SH3PXD2B | Up-regulated |
| SLC12A7 | Down-regulated | DNAJC12 | Up-regulated |
| ARHGAP18 | Down-regulated | AEBP1 | Up-regulated |
| ITGA11 | Down-regulated | RP6-33F8.1 | Up-regulated |
| LST1 | Down-regulated | TBX2 | Up-regulated |
| FNBP1 | Down-regulated | LOXL1-AS1 | Up-regulated |
| RP11-148B3.1 | Down-regulated | NFE4 | Up-regulated |
| RP11-282O18.3 | Down-regulated | PAK6 | Up-regulated |
| SLCO3A1 | Down-regulated | CD19 | Up-regulated |
| CTSW | Down-regulated | FKBP14 | Up-regulated |
| KIF6 | Down-regulated | RP11-44N11.2 | Up-regulated |
| ICAM3 | Down-regulated | CATSPERB | Up-regulated |
| VCL | Down-regulated | SNPH | Up-regulated |
| HLX | Down-regulated | AKAP12 | Up-regulated |
| MPEG1 | Down-regulated | COL9A3 | Up-regulated |
| MAPKBP1 | Down-regulated | VGLL3 | Up-regulated |
| KCTD5 | Down-regulated | SLC26A10 | Up-regulated |
| PLD4 | Down-regulated | TBX4 | Up-regulated |
| RP11-256I23.3 | Down-regulated | NRCAM | Up-regulated |
| ZNF185 | Down-regulated | AP001092.4 | Up-regulated |
| CTNNBIP1 | Down-regulated | BHMT2 | Up-regulated |
| JAK2 | Down-regulated | LOXL1 | Up-regulated |
| ECM1 | Down-regulated | CFH | Up-regulated |
| EPHA1-AS1 | Down-regulated | BCAM | Up-regulated |
| GCSAML | Down-regulated | EDN1 | Up-regulated |
| TLR6 | Down-regulated | JAG2 | Up-regulated |
| PREX1 | Down-regulated | REEP2 | Up-regulated |
| AR | Down-regulated | PCSK5 | Up-regulated |
| SNX10 | Down-regulated | RP11-44N11.1 | Up-regulated |
| RNASE2 | Down-regulated | CXCL12 | Up-regulated |
| LPAR1 | Down-regulated | TC2N | Up-regulated |
| RP11-376P6.3 | Down-regulated | LRRN1 | Up-regulated |
| TYROBP | Down-regulated | SBK3 | Up-regulated |
| MEF2D | Down-regulated | IGF1R | Up-regulated |
| GRN | Down-regulated | RP11-439E19.7 | Up-regulated |
| IL12A-AS1 | Down-regulated | DKK2 | Up-regulated |
| CBX6 | Down-regulated | INO80C | Up-regulated |
| IL13RA1 | Down-regulated | LRRC70 | Up-regulated |
| HOXB-AS1 | Down-regulated | RP11-125B21.2 | Up-regulated |
| CERK | Down-regulated | FBLN5 | Up-regulated |
| LRRC38 | Down-regulated | FAM153C | Up-regulated |
| SELL | Down-regulated | CXorf65 | Up-regulated |
| KCTD1 | Down-regulated | AGPAT4 | Up-regulated |
| PAPLN | Down-regulated | CEACAM8 | Up-regulated |
| PARVG | Down-regulated | MFSD10 | Up-regulated |
| PKIB | Down-regulated | CCR4 | Up-regulated |
| SLC2A12 | Down-regulated | FOXC2 | Up-regulated |
| SLC44A1 | Down-regulated | ST5 | Up-regulated |
| RXFP1 | Down-regulated | PLCL1 | Up-regulated |
| TNFRSF1B | Down-regulated | DAAM2 | Up-regulated |
| SDS | Down-regulated | PLCB4 | Up-regulated |
| REPIN1 | Down-regulated | SYNE2 | Up-regulated |
| ZFP36L2 | Down-regulated | PDGFD | Up-regulated |
| RP11-192P3.5 | Down-regulated | RASSF8-AS1 | Up-regulated |
| IRF8 | Down-regulated | ALPL | Up-regulated |
| RASSF2 | Down-regulated | OLFML2A | Up-regulated |
| NEGR1 | Down-regulated | CABP4 | Up-regulated |
| SRC | Down-regulated | HOMER1 | Up-regulated |
| EEPD1 | Down-regulated | C3orf70 | Up-regulated |
| DEFB1 | Down-regulated | CSF1 | Up-regulated |
| RP11-680F8.1 | Down-regulated | RP11-180P8.1 | Up-regulated |
| CYTH4 | Down-regulated | TDRD10 | Up-regulated |
| ARHGAP31 | Down-regulated | RP11-815I9.4 | Up-regulated |
| ALDH2 | Down-regulated | TRO | Up-regulated |
| SLC50A1 | Down-regulated | RP11-166D19.1 | Up-regulated |
| CPNE3 | Down-regulated | BMP4 | Up-regulated |
| RP4-647C14.2 | Down-regulated | COL6A1 | Up-regulated |
| ZC3H12C | Down-regulated | CXCL6 | Up-regulated |
| HTR1F | Down-regulated | RP11-326C3.7 | Up-regulated |
| STS | Down-regulated | PRRT4 | Up-regulated |
| CNNM4 | Down-regulated | CCL5 | Up-regulated |
| UNC93B1 | Down-regulated | CYSLTR2 | Up-regulated |
| RP11-490M8.1 | Down-regulated | PCBP4 | Up-regulated |
| RP11-375A5.1 | Down-regulated | LAMB4 | Up-regulated |
| PTGFRN | Down-regulated | RP11-324E6.9 | Up-regulated |
| TCTEX1D1 | Down-regulated | CHDH | Up-regulated |
| TNFSF8 | Down-regulated | RP11-1334A24.6 | Up-regulated |
| CXXC5 | Down-regulated | TGFBR3 | Up-regulated |
| AC007392.3 | Down-regulated | CNTN4-AS1 | Up-regulated |
| MOB3B | Down-regulated | CSRP2 | Up-regulated |
| MTMR1 | Down-regulated | EFS | Up-regulated |
| C15orf39 | Down-regulated | FTCD | Up-regulated |
| OLIG1 | Down-regulated | UGT2B28 | Up-regulated |
| RP11-439L8.4 | Down-regulated | LDLRAD3 | Up-regulated |
| PADI4 | Down-regulated | UBE2E3 | Up-regulated |
| ETS2 | Down-regulated | TINAGL1 | Up-regulated |
| AOAH | Down-regulated | NCAM1 | Up-regulated |
| KCNK17 | Down-regulated | NUAK1 | Up-regulated |
| RP13-895J2.2 | Down-regulated | DDAH1 | Up-regulated |
| PISD | Down-regulated | RP5-857K21.4 | Up-regulated |
| TMEM8B | Down-regulated | IL17RC | Up-regulated |
| DBI | Down-regulated | P2RX5 | Up-regulated |
| TGFB1 | Down-regulated | SHE | Up-regulated |
| GP9 | Down-regulated | C6orf164 | Up-regulated |
| ALCAM | Down-regulated | SLC25A27 | Up-regulated |
| LRRC26 | Down-regulated | RASGRF2 | Up-regulated |
| TICAM1 | Down-regulated | TNFSF11 | Up-regulated |

**Table 2. 43 key proteins.**

| **Protein** | **Regulation type** | **Main function in development and progress of AML** | **PMID** |
| --- | --- | --- | --- |
| JAK2 | Down-regulated | a target for anticancer therapy | 27508038 |
| TLR7 | Down-regulated | activate antitumor immunity | 19449004 |
| ITGB5 | Down-regulated | activate invasion and metastatic | 16838325 |
| JAG1 | Down-regulated | activate leukemia development | 22960397 |
| SPARC | Down-regulated | aggressive leukemia growth | 24590286 |
| TYROBP | Down-regulated | associated with the leukemic phenotype | 25928846 |
| MEIS1 | Down-regulated | blunted the efficacy of chemotherapy | 28054140 |
| LAT2 | Down-regulated | contribute to the pathogenesis of AML | 21488857 |
| MYH11 | Down-regulated | fusion gene | 27836890 |
| IL3RA | Down-regulated | immunotherapeutic target | 25891481 |
| CCL1 | Down-regulated | induced pronounced chemotaxis | 21439940 |
| CD9 | Down-regulated | negative correlation with prognosis | 12717686 |
| RNASE2 | Down-regulated | negative correlation with prognosis | 15084694 |
| ATP1B1 | Down-regulated | negative correlation with prognosis | 26506237 |
| HOXB4 | Down-regulated | negative correlation with prognosis | 27150986 |
| MN1 | Down-regulated | negative correlation with prognosis | 27321378 |
| MYH9 | Down-regulated | negative correlation with prognosis | 27437869 |
| CD47 | Down-regulated | negative correlation with prognosis; therapeutic antibody target | 19632179 |
| BEX4 | Down-regulated | oncogene | 26408910 |
| ETS2 | Down-regulated | oncogene | 22221250 |
| CBFA2T3 | Down-regulated | pathogenic gene | 28112737 |
| CSPG4 | Down-regulated | potential marker | 25706398 |
| CXXC5 | Down-regulated | potential therapeutic target | 25605239 |
| STS | Down-regulated | supports the growth of AML1-ETO cells | 26449661 |
| HOXB2 | Down-regulated | tumor suppressors | 26482852 |
| HOXB3 | Down-regulated | tumor suppressors | 26482852 |
| BMP4 | Up-regulated | activates hematopoietic differentiation programs | 12064918 |
| CXCL12 | Up-regulated | blunted the efficacy of chemotherapy | 23826077 |
| EDN1 | Up-regulated | blunted the efficacy of chemotherapy | 28042875 |
| ITGB4 | Up-regulated | blunted the efficacy of chemotherapy | 22295105 |
| PRAME | Up-regulated | candidate target for the immunotherapy | 23691459 |
| RUNX1T1 | Up-regulated | fusion gene | 28166825 |
| CD226 | Up-regulated | increase antitumor effects | 26588911 |
| IGF1R | Up-regulated | inhibit apoptosis | 25186968 |
| CCL5 | Up-regulated | inhibited T cell migration resulting in influencing T cell targeting immunotherapy | 16267679 |
| BMI1 | Up-regulated | negative correlation with prognosis | 25084695 |
| CD81 | Up-regulated | negative correlation with prognosis | 27566555 |
| CFH | Up-regulated | negative correlation with prognosis | 27473565 |
| MLLT4 | Up-regulated | negative correlation with prognosis | 23630019 |
| VEGFA | Up-regulated | negative correlation with prognosis | 17983459 |
| CTGF | Up-regulated | positively correlated with bone marrow blast count | 24989271 |
| DLL3 | Up-regulated | promote cell proliferation | 19302234 |
| ARID5B | Up-regulated | risk gene | 22422485 |

**Table 3**. 447 targets ranked by the degree of PPIs.

The table listed the 447 genes in descending order of the degree of them in the network.

| **Rank** | **Target Name** | **Number of PPIs** | **Rank** | **Target Name** | **Number of PPIs** |
| --- | --- | --- | --- | --- | --- |
| 1 | ALDH2 | 704 | 225 | KCTD1 | 20 |
| 2 | MYH9 | 649 | 226 | MSR1 | 20 |
| 3 | OBSL1 | 616 | 227 | PARVG | 20 |
| 4 | SRC | 604 | 228 | PLXDC2 | 20 |
| 5 | MYH11 | 518 | 229 | SELL | 20 |
| 6 | EIF5A2 | 443 | 230 | SLC43A3 | 20 |
| 7 | PLCG1 | 436 | 231 | ST5 | 20 |
| 8 | IDH3A | 435 | 232 | TGFBR3 | 20 |
| 9 | AR | 415 | 233 | ZHX2 | 20 |
| 10 | BMI1 | 402 | 234 | ANPEP | 19 |
| 11 | LPAR1 | 391 | 235 | BIK | 19 |
| 12 | MYO1C | 363 | 236 | DDIT4L | 19 |
| 13 | KCNMA1 | 321 | 237 | FBLN5 | 19 |
| 14 | FBXO32 | 312 | 238 | HOXB2 | 19 |
| 15 | SDS | 304 | 239 | LOXL1 | 19 |
| 16 | ATXN1 | 277 | 240 | SCPEP1 | 19 |
| 17 | LCP1 | 271 | 241 | SLAIN1 | 19 |
| 18 | BAG2 | 243 | 242 | ABTB1 | 18 |
| 19 | DBN1 | 240 | 243 | CSRP2 | 18 |
| 20 | VCL | 232 | 244 | ELF4 | 18 |
| 21 | CD81 | 229 | 245 | FAM84B | 18 |
| 22 | ZNF662 | 222 | 246 | PADI4 | 18 |
| 23 | CCL5 | 213 | 247 | TANC2 | 18 |
| 24 | CAV1 | 212 | 248 | BCL9 | 17 |
| 25 | B9D1 | 197 | 249 | COL9A3 | 17 |
| 26 | CCR4 | 196 | 250 | MIF4GD | 17 |
| 27 | ATP6V0A1 | 192 | 251 | PAPPA | 17 |
| 28 | CXCL12 | 192 | 252 | PLP2 | 17 |
| 29 | JAK2 | 192 | 253 | C15orf39 | 16 |
| 30 | ADRA2C | 191 | 254 | CLIP3 | 16 |
| 31 | APLN | 191 | 255 | CPNE7 | 16 |
| 32 | CXCL6 | 190 | 256 | CXXC5 | 16 |
| 33 | HCAR1 | 187 | 257 | MTMR1 | 16 |
| 34 | UBE2E3 | 187 | 258 | RASAL1 | 16 |
| 35 | HTR1F | 186 | 259 | SFMBT1 | 16 |
| 36 | ARF3 | 178 | 260 | TNFSF13 | 16 |
| 37 | PRKCD | 164 | 261 | VOPP1 | 16 |
| 38 | RAB3IP | 161 | 262 | AGPAT4 | 15 |
| 39 | TOP1MT | 142 | 263 | ALCAM | 15 |
| 40 | TGFB1 | 141 | 264 | DOK6 | 15 |
| 41 | KRT18 | 139 | 265 | GP9 | 15 |
| 42 | EDN1 | 135 | 266 | ORAI1 | 15 |
| 43 | KCTD5 | 133 | 267 | DNAJC12 | 14 |
| 44 | TNFRSF1B | 133 | 268 | DSE | 14 |
| 45 | LPAR4 | 132 | 269 | FGD6 | 14 |
| 46 | CYSLTR2 | 131 | 270 | NOV | 14 |
| 47 | FNBP1 | 128 | 271 | PIK3AP1 | 14 |
| 48 | LAT2 | 128 | 272 | PLEKHB1 | 14 |
| 49 | PISD | 128 | 273 | PTGFRN | 14 |
| 50 | FOXL1 | 126 | 274 | RPH3AL | 14 |
| 51 | PIK3R3 | 124 | 275 | SERPINF1 | 14 |
| 52 | HIP1R | 122 | 276 | TLR6 | 14 |
| 53 | CTNND1 | 111 | 277 | ACKR2 | 13 |
| 54 | KRT8 | 111 | 278 | CAPG | 13 |
| 55 | CRMP1 | 110 | 279 | FKBP14 | 13 |
| 56 | CAMK2B | 106 | 280 | MBOAT2 | 13 |
| 57 | MYO6 | 105 | 281 | NR6A1 | 13 |
| 58 | SYNJ2 | 104 | 282 | PLXNB3 | 13 |
| 59 | TRIM32 | 102 | 283 | TMEM184A | 13 |
| 60 | FLNC | 101 | 284 | WWC2 | 13 |
| 61 | MPG | 100 | 285 | ZFP36L2 | 13 |
| 62 | ADRM1 | 96 | 286 | AK5 | 12 |
| 63 | IGF1R | 96 | 287 | CD58 | 12 |
| 64 | RXFP1 | 96 | 288 | GPM6B | 12 |
| 65 | SNAI1 | 95 | 289 | PARD3B | 12 |
| 66 | CBX6 | 93 | 290 | RAP1GAP2 | 12 |
| 67 | HOMER1 | 93 | 291 | ARHGEF25 | 11 |
| 68 | ITGB5 | 93 | 292 | COL27A1 | 11 |
| 69 | AP1S2 | 91 | 293 | DAAM2 | 11 |
| 70 | NR5A1 | 90 | 294 | DENND6B | 11 |
| 71 | EVC2 | 89 | 295 | PLBD1 | 11 |
| 72 | ATP1B1 | 88 | 296 | PPM1J | 11 |
| 73 | MEIS1 | 87 | 297 | RGS10 | 11 |
| 74 | RASSF8 | 86 | 298 | SLC44A1 | 11 |
| 75 | GRN | 83 | 299 | ANXA3 | 10 |
| 76 | NCKAP1 | 83 | 300 | GLIPR2 | 10 |
| 77 | VEGFA | 83 | 301 | HOXB3 | 10 |
| 78 | NCAM1 | 81 | 302 | IGSF1 | 10 |
| 79 | TMEM25 | 77 | 303 | LAX1 | 10 |
| 80 | NRP1 | 76 | 304 | MEGF10 | 10 |
| 81 | BMPR2 | 75 | 305 | PLEKHA8 | 10 |
| 82 | CYP2E1 | 75 | 306 | SHD | 10 |
| 83 | MLLT4 | 74 | 307 | SULF2 | 10 |
| 84 | MEF2A | 72 | 308 | TBX2 | 10 |
| 85 | ITGB4 | 69 | 309 | ASIC1 | 9 |
| 86 | RUNX1T1 | 69 | 310 | CACHD1 | 9 |
| 87 | PCBP4 | 66 | 311 | CHDH | 9 |
| 88 | TICAM1 | 63 | 312 | COL23A1 | 9 |
| 89 | CD1B | 60 | 313 | DFNA5 | 9 |
| 90 | SLC38A1 | 60 | 314 | EBI3 | 9 |
| 91 | TYROBP | 60 | 315 | ICAM3 | 9 |
| 92 | PACSIN1 | 59 | 316 | IL13RA1 | 9 |
| 93 | CSF1 | 58 | 317 | IQSEC2 | 9 |
| 94 | HS3ST3A1 | 57 | 318 | SH3PXD2B | 9 |
| 95 | INO80C | 57 | 319 | ZC3H12C | 9 |
| 96 | RERE | 57 | 320 | AIM1 | 8 |
| 97 | SYNE2 | 57 | 321 | FAM213A | 8 |
| 98 | CDK2AP1 | 56 | 322 | LDLRAD3 | 8 |
| 99 | KALRN | 56 | 323 | LILRA4 | 8 |
| 100 | LPIN3 | 56 | 324 | MOB3B | 8 |
| 101 | CHST3 | 55 | 325 | NFE4 | 8 |
| 102 | KIF21A | 55 | 326 | PKIB | 8 |
| 103 | WDR35 | 54 | 327 | POU4F1 | 8 |
| 104 | ARHGEF11 | 53 | 328 | PTPRN2 | 8 |
| 105 | MMP2 | 53 | 329 | SERPINE2 | 8 |
| 106 | LAMB3 | 52 | 330 | SGSH | 8 |
| 107 | EFHC2 | 51 | 331 | SLC12A7 | 8 |
| 108 | RASSF2 | 51 | 332 | SLC25A27 | 8 |
| 109 | SCHIP1 | 51 | 333 | SLC50A1 | 8 |
| 110 | TEAD1 | 51 | 334 | STAB1 | 8 |
| 111 | ANXA11 | 50 | 335 | CCL18 | 7 |
| 112 | ARHGAP21 | 50 | 336 | CDC42BPG | 7 |
| 113 | MEF2D | 50 | 337 | CLDN12 | 7 |
| 114 | CBFA2T3 | 49 | 338 | EMID1 | 7 |
| 115 | ARHGEF12 | 48 | 339 | EVC | 7 |
| 116 | CERK | 48 | 340 | ITGA11 | 7 |
| 117 | DTX4 | 48 | 341 | PTPRM | 7 |
| 118 | ETS2 | 48 | 342 | RHBDF1 | 7 |
| 119 | NDST3 | 48 | 343 | SASH3 | 7 |
| 120 | PLCB4 | 48 | 344 | SMPDL3B | 7 |
| 121 | RASGRF2 | 47 | 345 | TSPAN15 | 7 |
| 122 | DPYSL3 | 46 | 346 | BMP5 | 6 |
| 123 | HPCAL1 | 46 | 347 | CABP4 | 6 |
| 124 | PRAME | 46 | 348 | CD226 | 6 |
| 125 | RPS6KA2 | 46 | 349 | CNNM4 | 6 |
| 126 | SPARC | 45 | 350 | CRYBG3 | 6 |
| 127 | CD9 | 44 | 351 | EEPD1 | 6 |
| 128 | DBI | 43 | 352 | FAM101B | 6 |
| 129 | VLDLR | 43 | 353 | GLCCI1 | 6 |
| 130 | CPVL | 42 | 354 | HDX | 6 |
| 131 | BMP4 | 41 | 355 | MFSD10 | 6 |
| 132 | CSPG4 | 41 | 356 | MYO18B | 6 |
| 133 | IL3RA | 41 | 357 | NT5DC3 | 6 |
| 134 | IRF5 | 40 | 358 | PDGFD | 6 |
| 135 | PTPRB | 40 | 359 | RHBDL3 | 6 |
| 136 | AKAP12 | 39 | 360 | RTKN2 | 6 |
| 137 | JMY | 39 | 361 | SLC1A4 | 6 |
| 138 | PLD3 | 38 | 362 | SUSD1 | 6 |
| 139 | FOXC2 | 37 | 363 | TNFAIP2 | 6 |
| 140 | ROBO1 | 37 | 364 | BCAM | 5 |
| 141 | BIRC7 | 36 | 365 | ENPP4 | 5 |
| 142 | CD19 | 36 | 366 | IL17RC | 5 |
| 143 | CROCC | 36 | 367 | ITM2A | 5 |
| 144 | GPR183 | 36 | 368 | BEX4 | 5 |
| 145 | CLNK | 35 | 369 | NEGR1 | 5 |
| 146 | ALPL | 34 | 370 | PODXL2 | 5 |
| 147 | BTG2 | 34 | 371 | SHE | 5 |
| 148 | DOCK6 | 34 | 372 | SPINK2 | 5 |
| 149 | MAFK | 34 | 373 | SRPX | 5 |
| 150 | MAPKBP1 | 34 | 374 | AEBP1 | 4 |
| 151 | PELI2 | 34 | 375 | BHMT2 | 4 |
| 152 | SH3BP2 | 34 | 376 | BMP3 | 4 |
| 153 | TNFSF11 | 34 | 377 | CATSPERB | 4 |
| 154 | UNC93B1 | 34 | 378 | CEACAM8 | 4 |
| 155 | JAG1 | 33 | 379 | CRIM1 | 4 |
| 156 | MMP14 | 33 | 380 | FAM171A1 | 4 |
| 157 | NKD2 | 33 | 381 | GJA4 | 4 |
| 158 | NUAK1 | 33 | 382 | KIF6 | 4 |
| 159 | OLIG1 | 33 | 383 | MGLL | 4 |
| 160 | PREX1 | 33 | 384 | NRBP2 | 4 |
| 161 | STARD13 | 33 | 385 | RBFOX3 | 4 |
| 162 | CD47 | 32 | 386 | REEP2 | 4 |
| 163 | IL5RA | 32 | 387 | SNX10 | 4 |
| 164 | RAB3C | 31 | 388 | SPNS3 | 4 |
| 165 | ADA | 30 | 389 | ACSM3 | 3 |
| 166 | CDC42BPB | 30 | 390 | ELOVL7 | 3 |
| 167 | COBLL1 | 30 | 391 | FTCD | 3 |
| 168 | MCF2L | 30 | 392 | LCT | 3 |
| 169 | CACNA2D2 | 29 | 393 | LRP3 | 3 |
| 170 | COL6A2 | 29 | 394 | RANBP17 | 3 |
| 171 | EFS | 29 | 395 | RNASE2 | 3 |
| 172 | IRF8 | 29 | 396 | TCN1 | 3 |
| 173 | NRCAM | 29 | 397 | ZNF185 | 3 |
| 174 | PRTFDC1 | 29 | 398 | ZNF358 | 3 |
| 175 | SERPINE1 | 29 | 399 | BEX4 | 2 |
| 176 | SLC16A1 | 29 | 400 | C20orf203 | 2 |
| 177 | CDK14 | 28 | 401 | CD1E | 2 |
| 178 | FOXP4 | 28 | 402 | CTSW | 2 |
| 179 | HOXB4 | 28 | 403 | DEFB1 | 2 |
| 180 | JAG2 | 28 | 404 | DLL3 | 2 |
| 181 | SNX30 | 28 | 405 | EGFL6 | 2 |
| 182 | ARID5B | 26 | 406 | ILDR2 | 2 |
| 183 | CFH | 26 | 407 | LRRN1 | 2 |
| 184 | COL6A1 | 26 | 408 | LST1 | 2 |
| 185 | CYTH4 | 26 | 409 | MPEG1 | 2 |
| 186 | GSR | 26 | 410 | OLFML2A | 2 |
| 187 | ITGB7 | 26 | 411 | P2RX5 | 2 |
| 188 | PAPLN | 26 | 412 | PRRT4 | 2 |
| 189 | PLXNB2 | 26 | 413 | RNF112 | 2 |
| 190 | REPIN1 | 26 | 414 | SLC2A12 | 2 |
| 191 | COL14A1 | 25 | 415 | SLC4A3 | 2 |
| 192 | CPNE3 | 25 | 416 | SLC7A10 | 2 |
| 193 | ECM1 | 25 | 417 | STS | 2 |
| 194 | EMILIN1 | 25 | 418 | TBX4 | 2 |
| 195 | PCSK5 | 25 | 419 | TMEM8B | 2 |
| 196 | SELE | 25 | 420 | TNFSF8 | 2 |
| 197 | TNFRSF11A | 25 | 421 | WDFY4 | 2 |
| 198 | ARHGAP18 | 24 | 422 | ACSM1 | 1 |
| 199 | C20orf195 | 24 | 423 | AOAH | 1 |
| 200 | CTNNBIP1 | 24 | 424 | C2orf54 | 1 |
| 201 | HLX | 24 | 425 | CCL1 | 1 |
| 202 | KCNK16 | 24 | 426 | CECR6 | 1 |
| 203 | LHX6 | 24 | 427 | DKK2 | 1 |
| 204 | SIPA1L2 | 24 | 428 | DNAH8 | 1 |
| 205 | TLR7 | 24 | 429 | EMP2 | 1 |
| 206 | TRO | 24 | 430 | ERVFRD-1 | 1 |
| 207 | MAGIX | 23 | 431 | IGSF10 | 1 |
| 208 | PCCA | 23 | 432 | KCNK17 | 1 |
| 209 | TANC1 | 23 | 433 | KIF25-AS1 | 1 |
| 210 | TNS3 | 23 | 434 | LAMB4 | 1 |
| 211 | ACP6 | 22 | 435 | LRRC26 | 1 |
| 212 | MUSK | 22 | 436 | LRRC70 | 1 |
| 213 | PAK6 | 22 | 437 | MYRF | 1 |
| 214 | WIPF3 | 22 | 438 | PLCL1 | 1 |
| 215 | ARHGAP31 | 21 | 439 | PLD4 | 1 |
| 216 | CPE | 21 | 440 | PQLC2 | 1 |
| 217 | CUEDC1 | 21 | 441 | RD3L | 1 |
| 218 | GSTM2 | 21 | 442 | SLCO3A1 | 1 |
| 219 | IL17RA | 21 | 443 | SNPH | 1 |
| 220 | PALM | 21 | 444 | TC2N | 1 |
| 221 | TINAGL1 | 21 | 445 | TCTEX1D1 | 1 |
| 222 | UGT2B28 | 21 | 446 | TMEM44 | 1 |
| 223 | CTGF | 20 | 447 | VGLL3 | 1 |
| 224 | DDAH1 | 20 |  |  |  |

**Table 4.** 415 selected CM.

415 CM selected from TCMID and classified into 8 categories.

| **CM Name** | **category** | **CM Name** | **category** | **CM Name** | **category** |
| --- | --- | --- | --- | --- | --- |
| Elsholtzia ciliata | exterior-releasing herbs | semen holarrhenae | heat-clearing Herbs | Pyrola calliantha | antirheumatics |
| Mentha haplocalyx | exterior-releasing herbs | Herba Sarcatidrae | heat-clearing Herbs | Clerodendron cyrtophyllum | antirheumatics |
| Fructus Xanthii sibirici | exterior-releasing herbs | Amorpha fruticosa | heat-clearing Herbs | Tylophora ovata | antirheumatics |
| Bupleurum chinense | exterior-releasing herbs | Echinacea purpurea | heat-clearing Herbs | Siegesbeckia orientalis var. glabrescens | antirheumatics |
| Periostracum Cicadae | exterior-releasing herbs | Oxalis corniculata | heat-clearing Herbs | Veratrum grandiflorum | antirheumatics |
| Tamarix chinensis | exterior-releasing herbs | Colla Corii Asini | tonic herbs | Dioscorea septemloba | antirheumatics |
| Semen Sojae Praeparata | exterior-releasing herbs | Artemisia argyi | tonic herbs | Cocculus indicus | antirheumatics |
| Centipeda minima | exterior-releasing herbs | Bletilla striata | tonic herbs | Chaenomeles sinensis | antirheumatics |
| Saposhnikovia divaricata | exterior-releasing herbs | Veratrum album | tonic herbs | Loranthus parasiticus | antirheumatics |
| Ligusticum sinense | exterior-releasing herbs | Paeonia albiflora | tonic herbs | Pyrrosia lingua | antirheumatics |
| Pueraria lobata | exterior-releasing herbs | Glehnia littoralis | tonic herbs | Semen Cucumis | antirheumatics |
| Cinnamomum cassia | exterior-releasing herbs | Fructus Schiandrae | tonic herbs | Pygeum topengii | antirheumatics |
| Schizonepeta tenuifolia | exterior-releasing herbs | Carapax Trionycis | tonic herbs | Clematis chinensis | antirheumatics |
| Ephedra sinica | exterior-releasing herbs | Spinacia oleracea | tonic herbs | Xanthoceras sorbifolia | antirheumatics |
| Vitex trifolia | exterior-releasing herbs | Dipsacus asperoides | tonic herbs | Cacumen Tamaricis | antirheumatics |
| Equisetum hiemale | exterior-releasing herbs | Rosa roxburghii | tonic herbs | Thlaspi arvense | antirheumatics |
| Arctium lappa | exterior-releasing herbs | Allium sativum | tonic herbs | Herba Plantaginis minutae | antirheumatics |
| Notopterygium incisum | exterior-releasing herbs | Ziziphus jujuba | tonic herbs | Cynanchum paniculatum | antirheumatics |
| Cimicifuga dahurica | exterior-releasing herbs | Angelica sinensis | tonic herbs | Strophanthus divaricatus | antirheumatics |
| Zingiber officinale | exterior-releasing herbs | Codonopsis pilosula | tonic herbs | Achillea millefolium | antirheumatics |
| Elsholtzia splendens | exterior-releasing herbs | Rehmannia glutinosa | tonic herbs | Coix lacryma- jobi var. ma- yuen | antirheumatics |
| Eucalyptus globulus | heat-clearing Herbs | Syzygium aromaticum | tonic herbs | Herba Artemisiae Scopariae | antirheumatics |
| Peucedanum praeruptorum | heat-clearing Herbs | Cordyceps sinensis | tonic herbs | Artemisia capillaris | antirheumatics |
| Oldenlandia diffusa | heat-clearing Herbs | Semen Benincasae | tonic herbs | Pterospermum lanceaefolium | antirheumatics |
| Michelia alba | heat-clearing Herbs | Eucommia ulmoides | tonic herbs | Cinnamomum camphora | antirheumatics |
| Stephania cepharantha | heat-clearing Herbs | Taxus brevifolia | tonic herbs | Pinellia pedatisecta | antirheumatics |
| Patrinia villosa | heat-clearing Herbs | Apis mellifera ligustica | tonic herbs | Rhododendron micranthum | antirheumatics |
| Herba Patriniae | heat-clearing Herbs | Aconitum carmichaeli | tonic herbs | Imperata cylindrica var. major | hemostyptic |
| Isatis indigotica | heat-clearing Herbs | Rubus idaeus | tonic herbs | Thuja orientalis | hemostyptic |
| Ricinus communis | heat-clearing Herbs | Glycyrrhiza uralensis | tonic herbs | Rubus alceaefolius | hemostyptic |
| Polygonum aviculare | heat-clearing Herbs | Pueraria thomsonii | tonic herbs | Herba Cirsii Japonici | hemostyptic |
| Camellia sinensis | heat-clearing Herbs | Alpinia officinarum | tonic herbs | Euphorbia humifusa | hemostyptic |
| Radix Paeoniae Rubra | heat-clearing Herbs | Ilex cornuta | tonic herbs | Lamiophlomis rotata | hemostyptic |
| Semen Phaseoli | heat-clearing Herbs | Lycium chinense | tonic herbs | Diaphragma juglandis | hemostyptic |
| Herba Sedi sarmentosi | heat-clearing Herbs | Plastrum Testudinis | tonic herbs | flos puerariae lobatae | hemostyptic |
| Sagittaria sagittifolia | heat-clearing Herbs | Carapax et Plastrum Testudinis | tonic herbs | fructus Sophorae | hemostyptic |
| Rheum officinale | heat-clearing Herbs | Gecko | tonic herbs | Abelmoschus manihot | hemostyptic |
| Semen Sojae Germinatus | heat-clearing Herbs | Radix Pseudostellariae | tonic herbs | Typha latifolia | hemostyptic |
| Juncus effusus | heat-clearing Herbs | Holothuria leucospilota | tonic herbs | Strychnos nux-vomica | hemostyptic |
| Hypericum japonicum | heat-clearing Herbs | Triglochin maritimum | tonic herbs | Claviceps purpurea | hemostyptic |
| Fructus Kochiae | heat-clearing Herbs | Syngnathus | tonic herbs | Mangifera indica | hemostyptic |
| Cortex Lycii Radicis | heat-clearing Herbs | Calophyllum inophyllum | tonic herbs | Nelumbinis rhizomatis Preparata | hemostyptic |
| Benincasa hispida | heat-clearing Herbs | Terminalia chebula | tonic herbs | Typha angustata | hemostyptic |
| Thalictrum ichangense | heat-clearing Herbs | Polygonum multiflorum | tonic herbs | Fructus Syzygii | hemostyptic |
| Lycopersicon esculentum | heat-clearing Herbs | Semen Juglandis | tonic herbs | Solanum melongena | hemostyptic |
| Potentilla discolor | heat-clearing Herbs | Semen Glycine Max | tonic herbs | Panax pseudo- ginseng var. notoginseng | hemostyptic |
| Pteris multifida | heat-clearing Herbs | Sesamum indicum | tonic herbs | Diospyros kaki | hemostyptic |
| Pteris cretica var. nervosa | heat-clearing Herbs | Rhodiola crenulata | tonic herbs | Rhodomyrtus tomentosa | hemostyptic |
| Sedum lineare | heat-clearing Herbs | Radix Hedysari | tonic herbs | Rheum wittrocki | hemostyptic |
| Radix Glycyrrhizae uralensis | heat-clearing Herbs | Hericium erinaceus | tonic herbs | Senecio chrysanthemoides | hemostyptic |
| Herba Dracocephali tangutici | heat-clearing Herbs | Trigonella foenum -graecum | tonic herbs | Ipomoea aquatica | hemostyptic |
| Brassica oleracea var. capitata | heat-clearing Herbs | Testa Arachidis Hypogaeae | tonic herbs | Typha angustifolia | hemostyptic |
| Uncaria rhynchophylla | heat-clearing Herbs | Astragalus membranaceus | tonic herbs | Cirsium setosum | hemostyptic |
| Hypericum perforatum | heat-clearing Herbs | Reineckea carnea | tonic herbs | Syzygium samarangense | hemostyptic |
| Dysosma versipellis | heat-clearing Herbs | Curcuma longa | tonic herbs | Petiolus Trachycarpi | hemostyptic |
| Adenanthera pavonina | heat-clearing Herbs | Allium tuberosum | tonic herbs | Ligusticum chuanxiong | Hemorheologic agent |
| Camptotheca acuminata | heat-clearing Herbs | Ganoderma lucidum | tonic herbs | Leonurus heterophyllus | Hemorheologic agent |
| Trifolium pratense | heat-clearing Herbs | Cornu Cervi Pantotrichum | tonic herbs | Salvia miltiorrhiza | Hemorheologic agent |
| Uncaria sinensis | heat-clearing Herbs | spirulina | tonic herbs | Carthamus tinctorius | Hemorheologic agent |
| Symplocos chinensis | heat-clearing Herbs | Arachis hypogaea | tonic herbs | Caulis Millettiae; Caulis spatholobi | Hemorheologic agent |
| Sophora japonica | heat-clearing Herbs | Saussurea laniceps | tonic herbs | Fructus Polygoni Orientalis | Hemorheologic agent |
| Phellodendron amurense | heat-clearing Herbs | Changium smyrnioides | tonic herbs | Gardenia jasminoides var. grandiflora | Hemorheologic agent |
| Artemisia annua | heat-clearing Herbs | Eclipta prostrata | tonic herbs | Caesalpinia sappan | Hemorheologic agent |
| Abrus fruticulosus | heat-clearing Herbs | Citrus limon | tonic herbs | Prunus persica | Hemorheologic agent |
| Centella asiatica | heat-clearing Herbs | Fructus Ligustri lucidi | tonic herbs | Eupolyphaga seu Steleophaga | Hemorheologic agent |
| Rhizoma Fagopyri Cymosi | heat-clearing Herbs | Vitis vinifera | tonic herbs | Radix chuanxiong | Hemorheologic agent |
| Lonicera japonica | heat-clearing Herbs | Panax ginseng | tonic herbs | Stigma Croci | Hemorheologic agent |
| Herba Cichorii | heat-clearing Herbs | Cistanche deserticola | tonic herbs | Rosa chinensis | Hemorheologic agent |
| Tupistra chinensis | heat-clearing Herbs | Ootheca Mantidis | tonic herbs | Lycopus lucidus | Hemorheologic agent |
| Tagetes patula | heat-clearing Herbs | Fructus Mori | tonic herbs | Sorbaria sorbifolia | Hemorheologic agent |
| Sophora flavescens | heat-clearing Herbs | fructus hippophae | tonic herbs | Nitraria tangutorum | Hemorheologic agent |
| Sophora alopecuroides | heat-clearing Herbs | Semen Astragali Complanati | tonic herbs | Fructus Leonuri | Hemorheologic agent |
| Acanthus ilicifolius | heat-clearing Herbs | Cnidium monnieri | tonic herbs | Hippophae rhamnoides | Hemorheologic agent |
| pericarpium pyri | heat-clearing Herbs | Radix Rehmanniae | tonic herbs | Fructus Choerospondiatis | Hemorheologic agent |
| Rhododendron huianum | heat-clearing Herbs | Herba Dendrobii | tonic herbs | Murraya paniculata | Hemorheologic agent |
| Cornu Saigae Tataricae | heat-clearing Herbs | Tricholoma matsutake | tonic herbs | Scolopendra subspinipes mutilans | Hemorheologic agent |
| Solanum nigrum | heat-clearing Herbs | Folium Pini | tonic herbs | Ginkgo biloba | energen-regulating drugs |
| Aloe vera | heat-clearing Herbs | Setaria italica | tonic herbs | Prunus mume | energen-regulating drugs |
| Strychnos ignatii | heat-clearing Herbs | Ziziphus jujuba var. spinosa | tonic herbs | Aquilaria sinensis | energen-regulating drugs |
| Folium camelliae sinensis | heat-clearing Herbs | Fructus Amomi | tonic herbs | Chelidonium majus | energen-regulating drugs |
| herba meconpsis integrifoliae | heat-clearing Herbs | Cynomorium songaricum | tonic herbs | White Caulis Perillae | energen-regulating drugs |
| Verbena officinalis | heat-clearing Herbs | Asparagus cochinchinensis | tonic herbs | Alpinia katsumadai | energen-regulating drugs |
| Portulaca oleracea | heat-clearing Herbs | Pulvis Ferri | tonic herbs | Pericarpium Citri Reticulatae | energen-regulating drugs |
| Rhododendron ovatum | heat-clearing Herbs | Cuscuta chinensis | tonic herbs | Caulis Clematidis Armandii | energen-regulating drugs |
| Rhododendron dauricum | heat-clearing Herbs | Fructus Evodiae | tonic herbs | Vladimiria souliei | energen-regulating drugs |
| Verbascum thapsus | heat-clearing Herbs | Radix Codonopsis pilosulae | tonic herbs | Pericarpium Arecae | energen-regulating drugs |
| Folium Viticis Negundo | heat-clearing Herbs | Panax quinquefolium | tonic herbs | Citrus medica var. sarcodactylis | energen-regulating drugs |
| Oroxylum indicum | heat-clearing Herbs | Asarum sieboldii | tonic herbs | Pericarpium Trichosanthis | energen-regulating drugs |
| Bombax malabaricum | heat-clearing Herbs | Agrimonia pilosa var. japonica | tonic herbs | Concha Meretricis seu Cyclinae | energen-regulating drugs |
| Eriobotrya japonica | heat-clearing Herbs | Saussurea involucrata | tonic herbs | Albizzia julibrissin | energen-regulating drugs |
| Melilotus suaveolens | heat-clearing Herbs | Cocos nucifera | tonic herbs | Apocynum lancifolium | energen-regulating drugs |
| Fritillaria ussuriensis | heat-clearing Herbs | Alpinia oxyphylla | tonic herbs | Magnolia officinalis | energen-regulating drugs |
| Taraxacum mongolicum | heat-clearing Herbs | Tremlla fuciformis | tonic herbs | Succinus | energen-regulating drugs |
| Senecio scandens | heat-clearing Herbs | Ulmus pumila | tonic herbs | Foeniculum vulgare | energen-regulating drugs |
| Artemisia apiacea | heat-clearing Herbs | Syringa pinnafolia | tonic herbs | Fagopyrum tataricum | energen-regulating drugs |
| Vatica rassak | heat-clearing Herbs | Taxus yunnanensis | tonic herbs | Litchi chinensis | energen-regulating drugs |
| Herba Nerviliae | heat-clearing Herbs | Polysaccharidum Versicolor | tonic herbs | Apocynum venetum | energen-regulating drugs |
| Semen Abutili | heat-clearing Herbs | Radix Hedysari Preparata | tonic herbs | Hordeum vulgare | energen-regulating drugs |
| Trifolium repens | heat-clearing Herbs | Radix Ginseng Rubra | tonic herbs | Populus tomentosa | energen-regulating drugs |
| Radix Tetrastigmatis Hemsleyani | heat-clearing Herbs | Zanthoxylum planispinum | tonic herbs | Rosa rugosa | energen-regulating drugs |
| Morus alba | heat-clearing Herbs | human placenta | tonic herbs | Fructus Caryophylli | energen-regulating drugs |
| Diphylleia grayi | heat-clearing Herbs | Aster tataricus | tonic herbs | Oleum Viticis Negundo | energen-regulating drugs |
| Phytolacca esculenta | heat-clearing Herbs | Phaseolus vulgaris | antirheumatics | Hibiscus syriacus | energen-regulating drugs |
| Flos Lonicerae | heat-clearing Herbs | Rhizoma Typhonii | antirheumatics | Saussurea lappa | energen-regulating drugs |
| Silybum marianum | heat-clearing Herbs | Cleome gynandra | antirheumatics | Fructus Terminaliae Preparata | energen-regulating drugs |
| Saussurea medusa | heat-clearing Herbs | Sinapis alba | antirheumatics | Gomphrena globosa | energen-regulating drugs |
| Bubalus bubalis | heat-clearing Herbs | rhizoma hterosmilacis | antirheumatics | Angelica decursiva | energen-regulating drugs |
| Salix purpurea | heat-clearing Herbs | Pinellia ternata | antirheumatics | Fagopyrum esculentum | energen-regulating drugs |
| Retinervus Luffae Fructus | heat-clearing Herbs | Dioscorea hypoglauca | antirheumatics | Aristolochia debilis | energen-regulating drugs |
| Pix Pini | heat-clearing Herbs | Bufo bufo gargarizans, Bufo melanostictus | antirheumatics | Pericarpium Citri Reticulatae Viride | energen-regulating drugs |
| Rheum tanguticum | heat-clearing Herbs | Radix Cudraniae | antirheumatics | Cephalotaxus fortunei | energen-regulating drugs |
| Herba Acalyphae | heat-clearing Herbs | Acanthopanax senticosus | antirheumatics | Amomum villosum | energen-regulating drugs |
| Rhizoma Bolbostemmae | heat-clearing Herbs | Hippophae rhamnoides | antirheumatics | Caulis Perillae Frutescentis | energen-regulating drugs |
| Smilax glabra | heat-clearing Herbs | Semen Malvae verticillatae | antirheumatics | Fructus Perillae | energen-regulating drugs |
| Potentilla chinensis | heat-clearing Herbs | Typhonium giganteum | antirheumatics | pericarpium papaveris | energen-regulating drugs |
| Ficus carica | heat-clearing Herbs | Rhizoma Pinelliae Preparata | antirheumatics | Citrus wilsonii | energen-regulating drugs |
| Pyrus communis | heat-clearing Herbs | Smilax menispermoidea | antirheumatics | Inula britannica | energen-regulating drugs |
| Rabdosia serra | heat-clearing Herbs | Semen Torreyae | antirheumatics | Flos Inulae | energen-regulating drugs |
| Abrus precatorius | heat-clearing Herbs | Nidus Vespae | antirheumatics | Akebia quinata | energen-regulating drugs |
| Oleum Linderae | heat-clearing Herbs | Herba Speranskiae tuberculatae | antirheumatics | Perilla frutescens var. arguta | energen-regulating drugs |
| Matteuccia struthiopteris | heat-clearing Herbs | Exodermis Poria | antirheumatics | Theobroma cacao | others |
| Semen Sojae | heat-clearing Herbs | Trichosanthes kirilowii | antirheumatics | Gossypium herbaceum | others |
| Scrophularia ningpoensis | heat-clearing Herbs | Semen Trichosanthis | antirheumatics | Capsicum annuum | others |
| Brucea javanica | heat-clearing Herbs | Piper kadsura | antirheumatics | Quisqualis indica | others |
| Commelina communis | heat-clearing Herbs | Lygodium japonicum | antirheumatics | Massa Medicata Fermentata | others |
| Taraxacum officinale | heat-clearing Herbs | Aristolochia heterophylla | antirheumatics | Massa Fermentata Hordei Germinatus | others |
| Chrysanthemum indicum | heat-clearing Herbs | Pericarpium lagenariae | antirheumatics | Crataegus pinnatifida | others |
| radix vitis romanetii | heat-clearing Herbs | Polygonum cuspidatum | antirheumatics | Veratrum nigrum var. ussuriense | others |
| Rhododendron mucronulatum | heat-clearing Herbs | Fraxinus ornus | antirheumatics | Cucumis melo | others |
| Phyllanthus emblica | heat-clearing Herbs | Paederia scandens | antirheumatics | Pedicellus Melo | others |
| Houttuynia cordata | heat-clearing Herbs | Sinoadina racemosa | antirheumatics | Moschus moschiferus | others |
| Spina gleditsiae | heat-clearing Herbs | Bombyx Batryticatus | antirheumatics | Liquidambar orientalis | others |
| Rheum palmatum | heat-clearing Herbs | Brassica juncea | antirheumatics | Radix Seu Rhizoma Valerianae | others |
| Fritillaria verticillata var. thunbergii | heat-clearing Herbs | Rhizoma Cibotii | antirheumatics | Catharanthus roseus | others |
| Margarita | heat-clearing Herbs | Laminaria japonica | antirheumatics | Rauvolfia verticillata | others |
| caulis Rubi idaei | heat-clearing Herbs | Tripterygium wilfordii | antirheumatics | Poria | others |
| Concha Margaritifera Usta | heat-clearing Herbs | Radix Smilacis | antirheumatics | Crassostrea gigas | others |
| Anemarrhena asphodeloides | heat-clearing Herbs | Sulfur | antirheumatics | Dens Draconis Preparata | others |
| Gardenia jasminoides | heat-clearing Herbs | Salix babylonica | antirheumatics |  |  |

**Table 5.** The KEGG pathways involving the DEGs generated by DAVID.

| **KEGG Pathway** | **Counts of genes** | **P-Value** | **FDR** |
| --- | --- | --- | --- |
| Focal adhesion | 19.00 | 8.70E-06 | 1.80E-03 |
| ECM-receptor interaction | 11.00 | 9.90E-05 | 1.00E-02 |
| Rheumatoid arthritis | 10.00 | 5.40E-04 | 3.70E-02 |
| Cytokine-cytokine receptor interaction | 16.00 | 1.20E-03 | 6.20E-02 |
| Hematopoietic cell lineage | 9.00 | 1.90E-03 | 7.60E-02 |
| PI3K-Akt signaling pathway | 20.00 | 2.10E-03 | 7.10E-02 |
| Cell adhesion molecules (CAMs) | 11.00 | 4.60E-03 | 1.30E-01 |
| Chemokine signaling pathway | 12.00 | 1.10E-02 | 2.50E-01 |
| Adherens junction | 7.00 | 1.20E-02 | 2.40E-01 |
| Proteoglycans in cancer | 12.00 | 1.80E-02 | 3.20E-01 |
| Rap1 signaling pathway | 12.00 | 2.50E-02 | 3.80E-01 |
| Protein digestion and absorption | 7.00 | 3.00E-02 | 4.10E-01 |
| Pathways in cancer | 18.00 | 3.40E-02 | 4.20E-01 |
| HIF-1 signaling pathway | 7.00 | 4.70E-02 | 5.10E-01 |
| Inflammatory mediator regulation of TRP channels | 7.00 | 4.70E-02 | 5.10E-01 |
| Regulation of actin cytoskeleton | 11.00 | 5.60E-02 | 5.50E-01 |
| Hypertrophic cardiomyopathy (HCM) | 6.00 | 5.70E-02 | 5.40E-01 |
| Chagas disease (American trypanosomiasis) | 7.00 | 6.00E-02 | 5.30E-01 |
| TNF signaling pathway | 7.00 | 6.40E-02 | 5.40E-01 |
| Amoebiasis | 7.00 | 6.40E-02 | 5.40E-01 |
| Glycerolipid metabolism | 5.00 | 6.90E-02 | 5.40E-01 |
| Dilated cardiomyopathy | 6.00 | 7.40E-02 | 5.50E-01 |
| GnRH signaling pathway | 6.00 | 9.60E-02 | 6.40E-01 |

**Table 6.** 205 KEGG pathways involved in the 8 CM categories.

The table lists all the KEGG pathway involved in 8 CM categories: 1 stands for exterior-releasing herbs, 2 stands for heat-clearing herbs, 3 stands for tonic herbs, 4 stands for antirheumatics, 5 stands for hemostyptic, 6 stands for hemorheologic agent, 7 stands for energen-regulating drugs, 8 stands for others.

| **KEGG pathway** | **Categories** |
| --- | --- |
| 2-Oxocarboxylic acid metabolism | 1,2 |
| Acute myeloid leukemia | 3 |
| Adherens junction | 3 |
| Adipocytokine signaling pathway | 1,2,3,4,5,6,7,8 |
| Adrenergic signaling in cardiomyocytes | 1,2 |
| African trypanosomiasis | 1,2 |
| Alanine, aspartate and glutamate metabolism | 1,2,3,4,5,7 |
| Alcoholism | 1,2 |
| Aldosterone synthesis and secretion | 1,2 |
| Aldosterone-regulated sodium reabsorption | 3 |
| alpha-Linolenic acid metabolism | 3 |
| Alzheimer's disease | 1,2,5,6 |
| Aminoacyl-tRNA biosynthesis | 1,2,3,4,7 |
| Amoebiasis | 1,2 |
| Amphetamine addiction | 1,2 |
| Amyotrophic lateral sclerosis (ALS) | 1,2 |
| Androgen and estrogen metabolism | 3,4,7,8 |
| Apoptosis | 3,8 |
| Arachidonic acid metabolism | 1,2,3,4,5,6,7,8 |
| Arginine and proline metabolism | 1,2,4,5,7,8 |
| Arginine biosynthesis | 2 |
| Arrhythmogenic right ventricular cardiomyopathy (ARVC) | 2 |
| Ascorbate and aldarate metabolism | 1,2,3,4,5,7,8 |
| B cell receptor signaling pathway | 2,4 |
| Base excision repair | 5 |
| beta-Alanine metabolism | 1,2,3,4,5,6,7 |
| Bile secretion | 1,2 |
| Biosynthesis of amino acids | 1,2 |
| Biosynthesis of antibiotics | 1,2 |
| Biosynthesis of unsaturated fatty acids | 1 |
| Bladder cancer | 2,3,4,5,6 |
| Butanoate metabolism | 3 |
| Butirosin and neomycin biosynthesis | 2 |
| Caffeine metabolism | 2,3,8 |
| Calcium signaling pathway | 1,2,3,4,5,6,7,8 |
| cAMP signaling pathway | 1,2 |
| Carbohydrate digestion and absorption | 2 |
| Carbon metabolism | 1,2 |
| Cardiac muscle contraction | 1,2 |
| Cell cycle | 3 |
| Central carbon metabolism in cancer | 1,2 |
| cGMP-PKG signaling pathway | 1,2 |
| Chagas disease (American trypanosomiasis) | 1,2 |
| Chemical carcinogenesis | 1 |
| Chemokine signaling pathway | 1,2,3,4,5,6,7,8 |
| Choline metabolism in cancer | 1,2 |
| Cholinergic synapse | 1,2 |
| Chronic myeloid leukemia | 2 |
| Circadian entrainment | 1,2 |
| Citrate cycle (TCA cycle) | 1,2 |
| Cocaine addiction | 1,2 |
| Collecting duct acid secretion | 2 |
| Colorectal cancer | 2 |
| Complement and coagulation cascades | 1,2,3,4,5,6,7 |
| Cyanoamino acid metabolism | 1,2,7 |
| Cysteine and methionine metabolism | 1,2,3,4,5,7 |
| Cytokine-cytokine receptor interaction | 8 |
| Cytosolic DNA-sensing pathway | 3 |
| D-Glutamine and D-glutamate metabolism | 1,2 |
| Dilated cardiomyopathy | 1,2,3,4,5,6,7 |
| Dopaminergic synapse | 1,2 |
| Drug metabolism | 3,4,5,6,7,8 |
| Drug metabolism - cytochrome P450 | 1,2 |
| Drug metabolism - other enzymes | 1 |
| Endocrine and other factor-regulated calcium reabsorption | 1,2 |
| Endometrial cancer | 1,2 |
| Epithelial cell signaling in Helicobacter pylori infection | 2,3,4,5,6,7,8 |
| Epstein-Barr virus infection | 2 |
| ErbB signaling pathway | 2 |
| Estrogen signaling pathway | 1,2 |
| Ether lipid metabolism | 1,2 |
| Fat digestion and absorption | 1,2 |
| Fatty acid biosynthesis | 1,2 |
| Fatty acid degradation | 1,2 |
| Fatty acid metabolism | 1,2,3,4,5,6,7,8 |
| Fc epsilon RI signaling pathway | 1,3,4,5,6,7,8 |
| Fc gamma R-mediated phagocytosis | 3,5,7 |
| Folate biosynthesis | 2 |
| FoxO signaling pathway | 1,2 |
| GABAergic synapse | 2 |
| Galactose metabolism | 1,2,3,4,5,6,7,8 |
| Gap junction | 2 |
| Gastric acid secretion | 2 |
| Glioma | 1,2 |
| Glucagon signaling pathway | 1,2 |
| Glutamatergic synapse | 1,2 |
| Glutathione metabolism | 1,2,3,4,6,7 |
| Glycerolipid metabolism | 3 |
| Glycerophospholipid metabolism | 1,2,3,4,6,7,8 |
| Glycine, serine and threonine metabolism | 1,3,4,7,8 |
| Glycolysis / Gluconeogenesis | 2 |
| Glycosaminoglycan degradation | 1 |
| Glycosylphosphatidylinositol(GPI)-anchor biosynthesis | 1,2,4,6 |
| Glyoxylate and dicarboxylate metabolism | 1,2,3,4,5,6,7,8 |
| GnRH signaling pathway | 1,2 |
| HIF-1 signaling pathway | 2 |
| Histidine metabolism | 2 |
| Hypertrophic cardiomyopathy (HCM) | 1,2 |
| Inflammatory mediator regulation of TRP channels | 1,2 |
| Inositol phosphate metabolism | 1,2 |
| Insulin resistance | 1,2 |
| Insulin secretion | 1,2 |
| Insulin signaling pathway | 2 |
| Legionellosis | 2 |
| Leishmaniasis | 1 |
| Leukocyte transendothelial migration | 1,2,3,4,5,6,7,8 |
| Linoleic acid metabolism | 1,2 |
| Long-term depression | 1,2,3,4,5,6,7,8 |
| Long-term potentiation | 1,6 |
| Lysine degradation | 1,2,3,4,5,6,7 |
| MAPK signaling pathway | 2,3,4,5,6,7 |
| Melanogenesis | 3 |
| Melanoma | 1,2 |
| Metabolic pathways | 1,2 |
| Metabolism of xenobiotics by cytochrome P450 | 1,2,3,4,5,6,7,8 |
| Methane metabolism | 4,7 |
| Mineral absorption | 2 |
| Morphine addiction | 1,2 |
| mTOR signaling pathway | 3 |
| Natural killer cell mediated cytotoxicity | 3 |
| Neuroactive ligand-receptor interaction | 1,2,3,4,5,6,7,8 |
| Neurotrophin signaling pathway | 1,2,3,4,5,6,7,8 |
| Nicotinate and nicotinamide metabolism | 3 |
| Nicotine addiction | 1,2 |
| NOD-like receptor signaling pathway | 3 |
| Non-small cell lung cancer | 1,2,3,4,5,6 |
| Notch signaling pathway | 6 |
| One carbon pool by folate | 1,2,3,4 |
| Oocyte meiosis | 1,2 |
| Other glycan degradation | 2 |
| Ovarian steroidogenesis | 1,2 |
| Oxytocin signaling pathway | 1,2 |
| Pancreatic cancer | 2 |
| Pancreatic secretion | 1,2 |
| Pantothenate and CoA biosynthesis | 2 |
| Pathways in cancer | 1,2,3 |
| Pentose and glucuronate interconversions | 1 |
| Pentose phosphate pathway | 8 |
| Peroxisome | 1,2 |
| Pertussis | 1,2 |
| Phagosome | 2 |
| Phenylalanine metabolism | 1,2,3,4,5,6,7 |
| Phenylalanine, tyrosine and tryptophan biosynthesis | 1,2,4,6,7 |
| Phosphatidylinositol signaling system | 6 |
| Phototransduction | 1,2 |
| Platelet activation | 1,2 |
| Porphyrin and chlorophyll metabolism | 1,2 |
| PPAR signaling pathway | 2 |
| Primary bile acid biosynthesis | 2 |
| Prion diseases | 2 |
| Progesterone-mediated oocyte maturation | 1,2,3,4,5,6,7,8 |
| Prolactin signaling pathway | 1 |
| Propanoate metabolism | 1 |
| Prostate cancer | 2,3,5 |
| Proteasome | 3 |
| Proteoglycans in cancer | 1,2 |
| Purine metabolism | 2 |
| Pyrimidine metabolism | 2 |
| Pyruvate metabolism | 1,2,3,4,5,7,8 |
| Rap1 signaling pathway | 1,2 |
| Ras signaling pathway | 1,2 |
| Regulation of actin cytoskeleton | 3 |
| Regulation of lipolysis in adipocytes | 1,2 |
| Renal cell carcinoma | 1,2 |
| Renin secretion | 1,2 |
| Retinol metabolism | 1,2 |
| Retrograde endocannabinoid signaling | 1,2 |
| Rheumatoid arthritis | 2 |
| Riboflavin metabolism | 2 |
| Ribosome | 3 |
| RIG-I-like receptor signaling pathway | 3 |
| RNA polymerase | 3 |
| Salivary secretion | 1,2 |
| Selenoamino acid metabolism | 3,4,7 |
| Serotonergic synapse | 1,2 |
| Sphingolipid metabolism | 1,2 |
| Sphingolipid signaling pathway | 1,2 |
| Staphylococcus aureus infection | 1,2 |
| Starch and sucrose metabolism | 1 |
| Steroid biosynthesis | 1 |
| Steroid hormone biosynthesis | 1,2,3,4,7,8 |
| Sulfur metabolism | 2 |
| Synaptic vesicle cycle | 2 |
| Synthesis and degradation of ketone bodies | 1,5,6,7,8 |
| T cell receptor signaling pathway | 6 |
| Taste transduction | 1,2,3,4,5,6,7,8 |
| Taurine and hypotaurine metabolism | 1,2,3,7 |
| Terpenoid backbone biosynthesis | 2,7 |
| Thyroid cancer | 2 |
| Thyroid hormone signaling pathway | 1,2 |
| Thyroid hormone synthesis | 1,2 |
| TNF signaling pathway | 1 |
| Toll-like receptor signaling pathway | 3,8 |
| Tryptophan metabolism | 3 |
| Tuberculosis | 2 |
| Type II diabetes mellitus | 1,2,3,4,5,6,7,8 |
| Tyrosine metabolism | 3 |
| Ubiquinone and other terpenoid-quinone biosynthesis | 3 |
| Valine, leucine and isoleucine biosynthesis | 1,2,3,4,7 |
| Valine, leucine and isoleucine degradation | 7 |
| Vascular smooth muscle contraction | 1,2,3,4,5,6,7,8 |
| VEGF signaling pathway | 1,2,3,4,5,6,7,8 |
| Vibrio cholerae infection | 3,4,5,7 |
| Vitamin digestion and absorption | 1,2 |
| Wnt signaling pathway | 1 |

Table 7. Explicit action of the 5 CMs.

| Herbs | Proteins | Action |
| --- | --- | --- |
| Panax ginseng | BMI1 | reaction |
| Panax ginseng | ATP1B1 | catalysis |
| Panax ginseng | CCL5 | reaction |
| Panax ginseng | CD47 | reaction |
| Panax ginseng | CTGF | activation |
| Panax ginseng | CXCL12 | reaction |
| Panax ginseng | EDN1 | reaction |
| Panax ginseng | ETS2 | reaction |
| Panax ginseng | IGF1R | reaction |
| Panax ginseng | IGF1R | catalysis |
| Panax ginseng | ITGB5 | reaction, catalysis, binding |
| Panax ginseng | MYH11 | reaction, catalysis, binding |
| Panax ginseng | MYH9 | reaction |
| Panax ginseng | TLR7 | reaction, binding |
| Panax ginseng | TYROBP | reaction, catalysis |
| Cornu Cervi Pantotrichum | JAK2 | reaction, catalysis, inhibition |
| Cornu Cervi Pantotrichum | TLR7 | reaction, binding |
| Cornu Cervi Pantotrichum | TYROBP | reaction, catalysis |
| Cornu Cervi Pantotrichum | EDN1 | inhibition, reaction |
| Cornu Cervi Pantotrichum | MYH11 | reaction, catalysis, binding |
| Cornu Cervi Pantotrichum | IGF1R | reaction, catalysis |
| Cornu Cervi Pantotrichum | ITGB5 | reaction, catalysis, binding |
| Cornu Cervi Pantotrichum | ATP1B1 | catalysis |
| Cornu Cervi Pantotrichum | MYH9 | reaction |
| Cornu Cervi Pantotrichum | VEGFA | activation |
| Cornu Cervi Pantotrichum | BMI1 | reaction |
| Cornu Cervi Pantotrichum | MYH9 | reaction |
| Cornu Cervi Pantotrichum | ATP1B1 | catalysis |
| Cornu Cervi Pantotrichum | CD47 | reaction |
| Cornu Cervi Pantotrichum | ETS2 | reaction |
| Ilex cornuta | CXCL12 | reaction |
| Ilex cornuta | CCL5 | reaction |
| Folium camelliae sinensis | CCL5 | reaction |
| Folium camelliae sinensis | CXCL12 | reaction |
| Camellia sinensis | HTR1F | reaction |
| Camellia sinensis | APLN | reaction |
| Camellia sinensis | LPAR1 | reaction |
| Camellia sinensis | CCL5 | reaction |
| Camellia sinensis | CCR4 | reaction |
| Camellia sinensis | HCAR1 | reaction |
| Camellia sinensis | ADRA2C | reaction |
| Camellia sinensis | CXCL6 | reaction |
| Camellia sinensis | CXCL12 | reaction |
| Camellia sinensis | VEGFA | inhibition |
| Camellia sinensis | APLN | reaction |
| Camellia sinensis | HTR1F | reaction |
| Camellia sinensis | LPAR1 | reaction |
| Camellia sinensis | CCL5 | reaction |
| Camellia sinensis | HCAR1 | reaction |
| Camellia sinensis | CXCL12 | reaction |

Table 8. CM pairs for inducing SL.

| **CM 1** | **CM 2** | **NO. AML-related SL pairs** | **NO.other SL pairs** |
| --- | --- | --- | --- |
| Camellia sinensis | Sinoadina racemosa | 2 | 0 |
| Camellia sinensis | Bupleurum chinense | 2 | 0 |
| Camellia sinensis | Symplocos chinensis | 2 | 0 |
| Camellia sinensis | Rhododendron ovatum | 2 | 0 |
| Camellia sinensis | Phyllanthus emblica | 2 | 0 |
| Camellia sinensis | Abelmoschus manihot | 2 | 0 |
| Camellia sinensis | Fructus Kochiae | 2 | 0 |
| Camellia sinensis | Typha angustifolia | 2 | 0 |
| Camellia sinensis | Astragalus membranaceus | 2 | 0 |
| Camellia sinensis | Pterospermum lanceaefolium | 2 | 0 |
| Camellia sinensis | Tamarix chinensis | 2 | 0 |
| Camellia sinensis | Radix Hedysari | 2 | 0 |
| Camellia sinensis | Mentha haplocalyx | 2 | 0 |
| Camellia sinensis | Colla Corii Asini | 2 | 0 |
| Camellia sinensis | Inula britannica | 2 | 0 |
| Camellia sinensis | Rhizoma Fagopyri Cymosi | 2 | 0 |
| Camellia sinensis | Aster tataricus | 2 | 0 |
| Camellia sinensis | Herba Artemisiae Scopariae | 2 | 0 |
| Camellia sinensis | Capsicum annuum | 2 | 0 |
| Camellia sinensis | Patrinia villosa | 2 | 0 |
| Camellia sinensis | Amomum villosum | 2 | 0 |
| Camellia sinensis | Semen Juglandis | 2 | 0 |
| Camellia sinensis | Imperata cylindrica var. major | 2 | 0 |
| Camellia sinensis | Brassica oleracea var. capitata | 2 | 0 |
| Camellia sinensis | Isatis indigotica | 2 | 0 |
| Camellia sinensis | Dioscorea hypoglauca | 2 | 0 |
| Camellia sinensis | Juncus effusus | 2 | 0 |
| Camellia sinensis | Rheum palmatum | 2 | 0 |
| Camellia sinensis | fructus Sophorae | 2 | 0 |
| Camellia sinensis | Scrophularia ningpoensis | 2 | 0 |
| Camellia sinensis | Herba Sedi sarmentosi | 2 | 0 |
| Camellia sinensis | Acanthopanax senticosus | 2 | 0 |
| Camellia sinensis | Pyrrosia lingua | 2 | 0 |
| Camellia sinensis | Rhododendron micranthum | 2 | 0 |
| Camellia sinensis | Fructus Xanthii sibirici | 2 | 0 |
| Camellia sinensis | Hericium erinaceus | 2 | 0 |
| Camellia sinensis | Chaenomeles sinensis | 2 | 0 |
| Camellia sinensis | Agrimonia pilosa var. japonica | 2 | 0 |
| Camellia sinensis | Senecio chrysanthemoides | 2 | 0 |
| Camellia sinensis | Herba Patriniae | 2 | 0 |
| Camellia sinensis | Flos Inulae | 2 | 0 |
| Camellia sinensis | Camptotheca acuminata | 2 | 0 |
| Camellia sinensis | Xanthoceras sorbifolia | 2 | 0 |
| Camellia sinensis | Homo sapiens | 2 | 0 |
| Camellia sinensis | Artemisia apiacea | 2 | 0 |
| Camellia sinensis | Reineckea carnea | 2 | 0 |
| Camellia sinensis | Pteris cretica var. Nervosa | 2 | 0 |
| Camellia sinensis | Cuscuta chinensis | 2 | 0 |
| Camellia sinensis | Herba Acalyphae | 2 | 0 |
| Camellia sinensis | Pyrola calliantha | 2 | 0 |
| Camellia sinensis | Ziziphus jujuba | 2 | 0 |
| Camellia sinensis | Artemisia capillaris | 2 | 0 |
| Camellia sinensis | Sedum lineare | 2 | 0 |
| Camellia sinensis | Folium Pini | 2 | 0 |
| Camellia sinensis | Piper kadsura | 2 | 0 |
| Camellia sinensis | Fructus Mori | 2 | 0 |
| Camellia sinensis | Typha angustata | 2 | 0 |
| Camellia sinensis | Radix Paeoniae Rubra | 2 | 0 |
| Camellia sinensis | Tagetes patula | 2 | 0 |
| Camellia sinensis | Loranthus parasiticus | 2 | 0 |
| Camellia sinensis | Rheum tanguticum | 2 | 0 |
| Camellia sinensis | Thuja orientalis | 2 | 0 |
| Camellia sinensis | Glehnia littoralis | 2 | 0 |
| Camellia sinensis | Fagopyrum tataricum | 2 | 0 |
| Camellia sinensis | Rheum officinale | 2 | 0 |
| Camellia sinensis | Citrus medica var. sarcodactylis | 2 | 0 |
| Camellia sinensis | Caulis Clematidis Armandii | 2 | 0 |
| Camellia sinensis | Rhododendron dauricum | 2 | 0 |
| Camellia sinensis | Fructus Ligustri lucidi | 2 | 0 |
| Camellia sinensis | Echinacea purpurea | 2 | 0 |
| Camellia sinensis | Apocynum lancifolium | 2 | 0 |
| Camellia sinensis | Thalictrum ichangense | 2 | 0 |
| Camellia sinensis | Fructus Caryophylli | 2 | 0 |
| Camellia sinensis | Hypericum japonicum | 2 | 0 |
| Camellia sinensis | Panax pseudo - ginseng var. notoginseng | 2 | 0 |
| Camellia sinensis | Pygeum topengii | 2 | 0 |
| Camellia sinensis | Equisetum hiemale | 2 | 0 |
| Camellia sinensis | Pericarpium Arecae | 2 | 0 |
| Camellia sinensis | Rhodiola crenulata | 2 | 0 |
| Camellia sinensis | Fructus Evodiae | 2 | 0 |
| Camellia sinensis | Rosa chinensis | 2 | 0 |
| Camellia sinensis | Arctium lappa | 2 | 0 |
| Camellia sinensis | Ginkgo biloba | 2 | 0 |
| Camellia sinensis | Rhododendron mucronulatum | 2 | 0 |
| Camellia sinensis | Syzygium samarangense | 2 | 0 |
| Camellia sinensis | Scolopendra subspinipes mutilans | 2 | 0 |
| Camellia sinensis | Caesalpinia sappan | 2 | 0 |
| Camellia sinensis | Trifolium pratense | 2 | 0 |
| Camellia sinensis | Artemisia annua | 2 | 0 |
| Camellia sinensis | Tremlla fuciformis | 2 | 0 |
| Camellia sinensis | Sorbaria sorbifolia | 2 | 0 |
| Camellia sinensis | Senecio scandens | 2 | 0 |
| Camellia sinensis | Carthamus tinctorius | 2 | 0 |
| Camellia sinensis | Nidus Vespae | 2 | 0 |
| Camellia sinensis | Eucommia ulmoides | 2 | 0 |
| Camellia sinensis | Rhodomyrtus tomentosa | 2 | 0 |
| Camellia sinensis | Dysosma versipellis | 2 | 0 |
| Camellia sinensis | Uncaria sinensis | 2 | 0 |
| Camellia sinensis | Cynomorium songaricum | 2 | 0 |
| Camellia sinensis | Centella asiatica | 2 | 0 |
| Camellia sinensis | Diaphragma juglandis | 2 | 0 |
| Camellia sinensis | Hippophae rhamnoides | 2 | 0 |
| Camellia sinensis | Solanum nigrum | 2 | 0 |
| Camellia sinensis | Polygonum aviculare | 2 | 0 |
| Camellia sinensis | Stephania cepharantha | 2 | 0 |
| Camellia sinensis | Sophora japonica | 2 | 0 |
| Camellia sinensis | Rubus idaeus | 2 | 0 |
| Camellia sinensis | Rhododendron huianum | 2 | 0 |
| Camellia sinensis | Ilex cornuta | 2 | 0 |
| Camellia sinensis | Paeonia albiflora | 2 | 0 |
| Camellia sinensis | Euphorbia humifusa | 2 | 0 |
| Camellia sinensis | Phellodendron amurense | 2 | 0 |
| Camellia sinensis | Sesamum indicum | 2 | 0 |
| Camellia sinensis | Apocynum venetum | 2 | 0 |
| Camellia sinensis | Diospyros kaki | 2 | 0 |
| Camellia sinensis | Potentilla chinensis | 2 | 0 |
| Camellia sinensis | Massa Medicata Fermentata | 2 | 0 |
| Camellia sinensis | Rhizoma Typhonii | 2 | 0 |
| Camellia sinensis | Cinnamomum cassia | 2 | 0 |
| Camellia sinensis | Trigonella foenum -graecum | 2 | 0 |
| Camellia sinensis | Houttuynia cordata | 2 | 0 |
| Camellia sinensis | Hypericum perforatum | 2 | 0 |
| Camellia sinensis | pericarpium pyri | 2 | 0 |
| Camellia sinensis | Typha latifolia | 2 | 0 |
| Camellia sinensis | Fructus Schiandrae | 2 | 0 |
| Camellia sinensis | Radix Pseudostellariae | 2 | 0 |
| Camellia sinensis | Cacumen Tamaricis | 2 | 0 |
| Camellia sinensis | Alpinia officinarum | 2 | 0 |
| Camellia sinensis | Asparagus cochinchinensis | 2 | 0 |
| Camellia sinensis | Taxus brevifolia | 2 | 0 |
| Camellia sinensis | Clerodendron cyrtophyllum | 2 | 0 |
| Camellia sinensis | Paederia scandens | 2 | 2 |
| Camellia sinensis | Semen Sojae Praeparata | 2 | 2 |
| Camellia sinensis | Herba Cichorii | 2 | 2 |
| Camellia sinensis | Taraxacum mongolicum | 2 | 2 |
| Camellia sinensis | Chrysanthemum indicum | 2 | 2 |
| Camellia sinensis | Angelica sinensis | 2 | 2 |
| Camellia sinensis | Gardenia jasminoides | 2 | 2 |
| Camellia sinensis | Curcuma longa | 2 | 2 |
| Camellia sinensis | Elsholtzia splendens | 2 | 2 |
| Camellia sinensis | Ephedra sinica | 2 | 2 |
| Camellia sinensis | Herba Dracocephali tangutici | 2 | 2 |
| Camellia sinensis | Fraxinus ornus | 2 | 2 |
| Camellia sinensis | Lygodium japonicum | 2 | 2 |
| Camellia sinensis | Achillea millefolium | 2 | 2 |
| Camellia sinensis | Periostracum Cicadae | 2 | 2 |
| Camellia sinensis | Artemisia argyi | 2 | 2 |
| Camellia sinensis | Centipeda minima | 2 | 2 |
| Camellia sinensis | Potentilla discolor | 2 | 2 |
| Camellia sinensis | Silybum marianum | 2 | 2 |
| Camellia sinensis | Pteris multifida | 2 | 2 |
| Camellia sinensis | Abrus precatorius | 2 | 2 |
| Camellia sinensis | Taraxacum officinale | 2 | 2 |
| Camellia sinensis | Fructus Syzygii | 2 | 2 |
| Camellia sinensis | Lamiophlomis rotata | 2 | 2 |
| Camellia sinensis | Apis mellifera ligustica | 2 | 2 |
| Camellia sinensis | herba meconpsis integrifoliae | 2 | 2 |
| Camellia sinensis | Commelina communis | 2 | 2 |
| Camellia sinensis | Saussurea medusa | 2 | 2 |
| Camellia sinensis | Fructus Choerospondiatis | 2 | 2 |
| Camellia sinensis | Acanthus ilicifolius | 2 | 2 |
| Camellia sinensis | Sagittaria sagittifolia | 2 | 2 |
| Camellia sinensis | Herba Dendrobii | 2 | 2 |
| Camellia sinensis | Radix Cudraniae | 2 | 3 |
| Camellia sinensis | Rheum wittrocki | 2 | 3 |
| Camellia sinensis | Smilax menispermoidea | 2 | 3 |
| Camellia sinensis | Testa Arachidis Hypogaeae | 2 | 3 |
| Camellia sinensis | Morus alba | 2 | 3 |
| Camellia sinensis | Veratrum album | 2 | 3 |
| Camellia sinensis | Smilax glabra | 2 | 3 |
| Camellia sinensis | Veratrum nigrum var. ussuriense | 2 | 3 |
| Camellia sinensis | Polygonum cuspidatum | 2 | 3 |
| Camellia sinensis | Vatica rassak | 2 | 3 |
| Camellia sinensis | Polygonum multiflorum | 2 | 3 |
| Camellia sinensis | Folium camelliae sinensis | 2 | 3 |
| Camellia sinensis | Veratrum grandiflorum | 2 | 3 |
| Camellia sinensis | Vitis vinifera | 2 | 3 |
| Camellia sinensis | Semen Phaseoli | 2 | 3 |
| Camellia sinensis | Herba Cirsii Japonici | 2 | 4 |
| Camellia sinensis | Typhonium giganteum | 2 | 4 |
| Camellia sinensis | Semen Astragali Complanati | 2 | 4 |
| Camellia sinensis | Concha Margaritifera Usta;Margarita | 2 | 4 |
| Camellia sinensis | Fagopyrum esculentum | 2 | 4 |
| Camellia sinensis | Citrus limon | 2 | 4 |
| Camellia sinensis | Cistanche deserticola | 2 | 4 |
| Camellia sinensis | Holothuria leucospilota | 2 | 4 |
| Camellia sinensis | Pericarpium Citri Reticulatae | 2 | 4 |
| Camellia sinensis | Coix lacryma- jobi var. ma - yuen | 2 | 4 |
| Camellia sinensis | caulis Rubi idaei | 2 | 4 |
| Camellia sinensis | Margarita | 2 | 4 |
| Camellia sinensis | Ootheca Mantidis | 2 | 4 |
| Camellia sinensis | spirulina | 2 | 5 |
| Camellia sinensis | Cornu Cervi Pantotrichum | 2 | 17 |
| Camellia sinensis | Panax ginseng | 2 | 17 |
| Cornu Cervi Pantotrichum | Herba Sarcatidrae | 1 | 0 |
| Cornu Cervi Pantotrichum | Herba Sedi sarmentosi | 1 | 0 |
| Cornu Cervi Pantotrichum | Herba Speranskiae tuberculatae | 1 | 0 |
| Cornu Cervi Pantotrichum | Hericium erinaceus | 1 | 0 |
| Cornu Cervi Pantotrichum | Hippophae rhamnoides | 1 | 0 |
| Cornu Cervi Pantotrichum | Homo sapiens | 1 | 0 |
| Cornu Cervi Pantotrichum | Hordeum vulgare | 1 | 0 |
| Cornu Cervi Pantotrichum | Houttuynia cordata | 1 | 0 |
| Cornu Cervi Pantotrichum | Taraxacum officinale | 1 | 0 |
| Cornu Cervi Pantotrichum | Taxus brevifolia | 1 | 0 |
| Cornu Cervi Pantotrichum | Thlaspi arvense | 1 | 0 |
| Cornu Cervi Pantotrichum | Thuja orientalis | 1 | 0 |
| Cornu Cervi Pantotrichum | Trichosanthes kirilowii | 1 | 0 |
| Cornu Cervi Pantotrichum | Herba Plantaginis minutae | 1 | 1 |
| Cornu Cervi Pantotrichum | Hypericum japonicum | 1 | 1 |
| Folium camelliae sinensis | Sinoadina racemosa | 2 | 0 |
| Folium camelliae sinensis | Bupleurum chinense | 2 | 0 |
| Folium camelliae sinensis | Symplocos chinensis | 2 | 0 |
| Folium camelliae sinensis | Rhododendron ovatum | 2 | 0 |
| Folium camelliae sinensis | Phyllanthus emblica | 2 | 0 |
| Folium camelliae sinensis | Abelmoschus manihot | 2 | 0 |
| Folium camelliae sinensis | Fructus Kochiae | 2 | 0 |
| Folium camelliae sinensis | Typha angustifolia | 2 | 0 |
| Folium camelliae sinensis | Astragalus membranaceus | 2 | 0 |
| Folium camelliae sinensis | Pterospermum lanceaefolium | 2 | 0 |
| Folium camelliae sinensis | Tamarix chinensis | 2 | 0 |
| Folium camelliae sinensis | Radix Hedysari | 2 | 0 |
| Folium camelliae sinensis | Mentha haplocalyx | 2 | 0 |
| Folium camelliae sinensis | Colla Corii Asini | 2 | 0 |
| Folium camelliae sinensis | Inula britannica | 2 | 0 |
| Folium camelliae sinensis | Rhizoma Fagopyri Cymosi | 2 | 0 |
| Folium camelliae sinensis | Aster tataricus | 2 | 0 |
| Folium camelliae sinensis | Herba Artemisiae Scopariae | 2 | 0 |
| Folium camelliae sinensis | Patrinia villosa | 2 | 0 |
| Folium camelliae sinensis | Amomum villosum | 2 | 0 |
| Folium camelliae sinensis | Semen Juglandis | 2 | 0 |
| Folium camelliae sinensis | Imperata cylindrica var. major | 2 | 0 |
| Folium camelliae sinensis | Brassica oleracea var. capitata | 2 | 0 |
| Folium camelliae sinensis | Isatis indigotica | 2 | 0 |
| Folium camelliae sinensis | Dioscorea hypoglauca | 2 | 0 |
| Folium camelliae sinensis | Juncus effusus | 2 | 0 |
| Folium camelliae sinensis | Rheum palmatum | 2 | 0 |
| Folium camelliae sinensis | fructus Sophorae | 2 | 0 |
| Folium camelliae sinensis | Scrophularia ningpoensis | 2 | 0 |
| Folium camelliae sinensis | Herba Sedi sarmentosi | 2 | 0 |
| Folium camelliae sinensis | Acanthopanax senticosus | 2 | 0 |
| Folium camelliae sinensis | Pyrrosia lingua | 2 | 0 |
| Folium camelliae sinensis | Rhododendron micranthum | 2 | 0 |
| Folium camelliae sinensis | Fructus Xanthii sibirici | 2 | 0 |
| Folium camelliae sinensis | Hericium erinaceus | 2 | 0 |
| Folium camelliae sinensis | Chaenomeles sinensis | 2 | 0 |
| Folium camelliae sinensis | Agrimonia pilosa var. japonica | 2 | 0 |
| Folium camelliae sinensis | Senecio chrysanthemoides | 2 | 0 |
| Folium camelliae sinensis | Herba Patriniae | 2 | 0 |
| Folium camelliae sinensis | Flos Inulae | 2 | 0 |
| Folium camelliae sinensis | Camptotheca acuminata | 2 | 0 |
| Folium camelliae sinensis | Xanthoceras sorbifolia | 2 | 0 |
| Folium camelliae sinensis | Homo sapiens | 2 | 0 |
| Folium camelliae sinensis | Artemisia apiacea | 2 | 0 |
| Folium camelliae sinensis | Reineckea carnea | 2 | 0 |
| Folium camelliae sinensis | Pteris cretica var. nervosa | 2 | 0 |
| Folium camelliae sinensis | Cuscuta chinensis | 2 | 0 |
| Folium camelliae sinensis | Herba Acalyphae | 2 | 0 |
| Folium camelliae sinensis | Pyrola calliantha | 2 | 0 |
| Folium camelliae sinensis | Ziziphus jujuba | 2 | 0 |
| Folium camelliae sinensis | Artemisia capillaris | 2 | 0 |
| Folium camelliae sinensis | Sedum lineare | 2 | 0 |
| Folium camelliae sinensis | Folium Pini | 2 | 0 |
| Folium camelliae sinensis | Piper kadsura | 2 | 0 |
| Folium camelliae sinensis | Fructus Mori | 2 | 0 |
| Folium camelliae sinensis | Typha angustata | 2 | 0 |
| Folium camelliae sinensis | Radix Paeoniae Rubra | 2 | 0 |
| Folium camelliae sinensis | Tagetes patula | 2 | 0 |
| Folium camelliae sinensis | Loranthus parasiticus | 2 | 0 |
| Folium camelliae sinensis | Rheum tanguticum | 2 | 0 |
| Folium camelliae sinensis | Thuja orientalis | 2 | 0 |
| Folium camelliae sinensis | Glehnia littoralis | 2 | 0 |
| Folium camelliae sinensis | Fagopyrum tataricum | 2 | 0 |
| Folium camelliae sinensis | Rheum officinale | 2 | 0 |
| Folium camelliae sinensis | Citrus medica var. sarcodactylis | 2 | 0 |
| Folium camelliae sinensis | Caulis Clematidis Armandii | 2 | 0 |
| Folium camelliae sinensis | Rhododendron dauricum | 2 | 0 |
| Folium camelliae sinensis | Fructus Ligustri lucidi | 2 | 0 |
| Folium camelliae sinensis | Echinacea purpurea | 2 | 0 |
| Folium camelliae sinensis | Apocynum lancifolium | 2 | 0 |
| Folium camelliae sinensis | Thalictrum ichangense | 2 | 0 |
| Folium camelliae sinensis | Fructus Caryophylli | 2 | 0 |
| Folium camelliae sinensis | Hypericum japonicum | 2 | 0 |
| Folium camelliae sinensis | Panax pseudo - ginseng var. notoginseng | 2 | 0 |
| Folium camelliae sinensis | Pygeum topengii | 2 | 0 |
| Folium camelliae sinensis | Equisetum hiemale | 2 | 0 |
| Folium camelliae sinensis | Pericarpium Arecae | 2 | 0 |
| Folium camelliae sinensis | Rhodiola crenulata | 2 | 0 |
| Folium camelliae sinensis | Fructus Evodiae | 2 | 0 |
| Folium camelliae sinensis | Rosa chinensis | 2 | 0 |
| Folium camelliae sinensis | Arctium lappa | 2 | 0 |
| Folium camelliae sinensis | Ginkgo biloba | 2 | 0 |
| Folium camelliae sinensis | Rhododendron mucronulatum | 2 | 0 |
| Folium camelliae sinensis | Syzygium samarangense | 2 | 0 |
| Folium camelliae sinensis | Scolopendra subspinipes mutilans | 2 | 0 |
| Folium camelliae sinensis | Caesalpinia sappan | 2 | 0 |
| Folium camelliae sinensis | Trifolium pratense | 2 | 0 |
| Folium camelliae sinensis | Artemisia annua | 2 | 0 |
| Folium camelliae sinensis | Tremlla fuciformis | 2 | 0 |
| Folium camelliae sinensis | Sorbaria sorbifolia | 2 | 0 |
| Folium camelliae sinensis | Senecio scandens | 2 | 0 |
| Folium camelliae sinensis | Carthamus tinctorius | 2 | 0 |
| Folium camelliae sinensis | Nidus Vespae | 2 | 0 |
| Folium camelliae sinensis | Eucommia ulmoides | 2 | 0 |
| Folium camelliae sinensis | Rhodomyrtus tomentosa | 2 | 0 |
| Folium camelliae sinensis | Dysosma versipellis | 2 | 0 |
| Folium camelliae sinensis | Uncaria sinensis | 2 | 0 |
| Folium camelliae sinensis | Cynomorium songaricum | 2 | 0 |
| Folium camelliae sinensis | Centella asiatica | 2 | 0 |
| Folium camelliae sinensis | Diaphragma juglandis | 2 | 0 |
| Folium camelliae sinensis | Hippophae rhamnoides | 2 | 0 |
| Folium camelliae sinensis | Solanum nigrum | 2 | 0 |
| Folium camelliae sinensis | Polygonum aviculare | 2 | 0 |
| Folium camelliae sinensis | Stephania cepharantha | 2 | 0 |
| Folium camelliae sinensis | Sophora japonica | 2 | 0 |
| Folium camelliae sinensis | Rubus idaeus | 2 | 0 |
| Folium camelliae sinensis | Rhododendron huianum | 2 | 0 |
| Folium camelliae sinensis | Ilex cornuta | 2 | 0 |
| Folium camelliae sinensis | Paeonia albiflora | 2 | 0 |
| Folium camelliae sinensis | Euphorbia humifusa | 2 | 0 |
| Folium camelliae sinensis | Phellodendron amurense | 2 | 0 |
| Folium camelliae sinensis | Sesamum indicum | 2 | 0 |
| Folium camelliae sinensis | Apocynum venetum | 2 | 0 |
| Folium camelliae sinensis | Potentilla chinensis | 2 | 0 |
| Folium camelliae sinensis | Massa Medicata Fermentata | 2 | 0 |
| Folium camelliae sinensis | Rhizoma Typhonii | 2 | 0 |
| Folium camelliae sinensis | Cinnamomum cassia | 2 | 0 |
| Folium camelliae sinensis | Trigonella foenum -graecum | 2 | 0 |
| Folium camelliae sinensis | Houttuynia cordata | 2 | 0 |
| Folium camelliae sinensis | Hypericum perforatum | 2 | 0 |
| Folium camelliae sinensis | pericarpium pyri | 2 | 0 |
| Folium camelliae sinensis | Typha latifolia | 2 | 0 |
| Folium camelliae sinensis | Fructus Schiandrae | 2 | 0 |
| Folium camelliae sinensis | Radix Pseudostellariae | 2 | 0 |
| Folium camelliae sinensis | Cacumen Tamaricis | 2 | 0 |
| Folium camelliae sinensis | Alpinia officinarum | 2 | 0 |
| Folium camelliae sinensis | Asparagus cochinchinensis | 2 | 0 |
| Folium camelliae sinensis | Taxus brevifolia | 2 | 0 |
| Folium camelliae sinensis | Clerodendron cyrtophyllum | 2 | 0 |
| Folium camelliae sinensis | Paederia scandens | 2 | 2 |
| Folium camelliae sinensis | Semen Sojae Praeparata | 2 | 2 |
| Folium camelliae sinensis | Herba Cichorii | 2 | 2 |
| Folium camelliae sinensis | Taraxacum mongolicum | 2 | 2 |
| Folium camelliae sinensis | Chrysanthemum indicum | 2 | 2 |
| Folium camelliae sinensis | Angelica sinensis | 2 | 2 |
| Folium camelliae sinensis | Gardenia jasminoides | 2 | 2 |
| Folium camelliae sinensis | Curcuma longa | 2 | 2 |
| Folium camelliae sinensis | Elsholtzia splendens | 2 | 2 |
| Folium camelliae sinensis | Ephedra sinica | 2 | 2 |
| Folium camelliae sinensis | Herba Dracocephali tangutici | 2 | 2 |
| Folium camelliae sinensis | Fraxinus ornus | 2 | 2 |
| Folium camelliae sinensis | Lygodium japonicum | 2 | 2 |
| Folium camelliae sinensis | Achillea millefolium | 2 | 2 |
| Folium camelliae sinensis | Periostracum Cicadae | 2 | 2 |
| Folium camelliae sinensis | Artemisia argyi | 2 | 2 |
| Folium camelliae sinensis | Centipeda minima | 2 | 2 |
| Folium camelliae sinensis | Potentilla discolor | 2 | 2 |
| Folium camelliae sinensis | Silybum marianum | 2 | 2 |
| Folium camelliae sinensis | Pteris multifida | 2 | 2 |
| Folium camelliae sinensis | Abrus precatorius | 2 | 2 |
| Folium camelliae sinensis | Taraxacum officinale | 2 | 2 |
| Folium camelliae sinensis | Fructus Syzygii | 2 | 2 |
| Folium camelliae sinensis | Lamiophlomis rotata | 2 | 2 |
| Folium camelliae sinensis | Apis mellifera ligustica | 2 | 2 |
| Folium camelliae sinensis | herba meconpsis integrifoliae | 2 | 2 |
| Folium camelliae sinensis | Commelina communis | 2 | 2 |
| Folium camelliae sinensis | Saussurea medusa | 2 | 2 |
| Folium camelliae sinensis | Fructus Choerospondiatis | 2 | 2 |
| Folium camelliae sinensis | Acanthus ilicifolius | 2 | 2 |
| Folium camelliae sinensis | Sagittaria sagittifolia | 2 | 2 |
| Folium camelliae sinensis | Herba Dendrobii | 2 | 2 |
| Folium camelliae sinensis | Radix Cudraniae | 2 | 3 |
| Folium camelliae sinensis | Rheum wittrocki | 2 | 3 |
| Folium camelliae sinensis | Smilax menispermoidea | 2 | 3 |
| Folium camelliae sinensis | Testa Arachidis Hypogaeae | 2 | 3 |
| Folium camelliae sinensis | Morus alba | 2 | 3 |
| Folium camelliae sinensis | Veratrum album | 2 | 3 |
| Folium camelliae sinensis | Smilax glabra | 2 | 3 |
| Folium camelliae sinensis | Veratrum nigrum var. ussuriense | 2 | 3 |
| Folium camelliae sinensis | Polygonum cuspidatum | 2 | 3 |
| Folium camelliae sinensis | Vatica rassak | 2 | 3 |
| Folium camelliae sinensis | Polygonum multiflorum | 2 | 3 |
| Folium camelliae sinensis | Veratrum grandiflorum | 2 | 3 |
| Folium camelliae sinensis | Vitis vinifera | 2 | 3 |
| Folium camelliae sinensis | Semen Phaseoli | 2 | 3 |
| Folium camelliae sinensis | Herba Cirsii Japonici | 2 | 4 |
| Folium camelliae sinensis | Typhonium giganteum | 2 | 4 |
| Folium camelliae sinensis | Semen Astragali Complanati | 2 | 4 |
| Folium camelliae sinensis | Concha Margaritifera Usta;Margarita | 2 | 4 |
| Folium camelliae sinensis | Fagopyrum esculentum | 2 | 4 |
| Folium camelliae sinensis | Citrus limon | 2 | 4 |
| Folium camelliae sinensis | Holothuria leucospilota | 2 | 4 |
| Folium camelliae sinensis | Pericarpium Citri Reticulatae | 2 | 4 |
| Folium camelliae sinensis | Coix lacryma- jobi var. ma - yuen | 2 | 4 |
| Folium camelliae sinensis | caulis Rubi idaei | 2 | 4 |
| Folium camelliae sinensis | Margarita | 2 | 4 |
| Folium camelliae sinensis | Ootheca Mantidis | 2 | 4 |
| Folium camelliae sinensis | spirulina | 2 | 5 |
| Folium camelliae sinensis | Cornu Cervi Pantotrichum | 2 | 17 |
| Folium camelliae sinensis | Panax ginseng | 2 | 17 |
| Ilex cornuta | Sinoadina racemosa | 2 | 0 |
| Ilex cornuta | Bupleurum chinense | 2 | 0 |
| Ilex cornuta | Symplocos chinensis | 2 | 0 |
| Ilex cornuta | Rhododendron ovatum | 2 | 0 |
| Ilex cornuta | Phyllanthus emblica | 2 | 0 |
| Ilex cornuta | Abelmoschus manihot | 2 | 0 |
| Ilex cornuta | Fructus Kochiae | 2 | 0 |
| Ilex cornuta | Typha angustifolia | 2 | 0 |
| Ilex cornuta | Astragalus membranaceus | 2 | 0 |
| Ilex cornuta | Pterospermum lanceaefolium | 2 | 0 |
| Ilex cornuta | Tamarix chinensis | 2 | 0 |
| Ilex cornuta | Radix Hedysari | 2 | 0 |
| Ilex cornuta | Mentha haplocalyx | 2 | 0 |
| Ilex cornuta | Colla Corii Asini | 2 | 0 |
| Ilex cornuta | Inula britannica | 2 | 0 |
| Ilex cornuta | Rhizoma Fagopyri Cymosi | 2 | 0 |
| Ilex cornuta | Aster tataricus | 2 | 0 |
| Ilex cornuta | Herba Artemisiae Scopariae | 2 | 0 |
| Ilex cornuta | Patrinia villosa | 2 | 0 |
| Ilex cornuta | Amomum villosum | 2 | 0 |
| Ilex cornuta | Semen Juglandis | 2 | 0 |
| Ilex cornuta | Imperata cylindrica var. major | 2 | 0 |
| Ilex cornuta | Brassica oleracea var. capitata | 2 | 0 |
| Ilex cornuta | Isatis indigotica | 2 | 0 |
| Ilex cornuta | Dioscorea hypoglauca | 2 | 0 |
| Ilex cornuta | Juncus effusus | 2 | 0 |
| Ilex cornuta | Rheum palmatum | 2 | 0 |
| Ilex cornuta | Scrophularia ningpoensis | 2 | 0 |
| Ilex cornuta | Herba Sedi sarmentosi | 2 | 0 |
| Ilex cornuta | Acanthopanax senticosus | 2 | 0 |
| Ilex cornuta | Pyrrosia lingua | 2 | 0 |
| Ilex cornuta | Rhododendron micranthum | 2 | 0 |
| Ilex cornuta | Fructus Xanthii sibirici | 2 | 0 |
| Ilex cornuta | Hericium erinaceus | 2 | 0 |
| Ilex cornuta | Chaenomeles sinensis | 2 | 0 |
| Ilex cornuta | Agrimonia pilosa var. japonica | 2 | 0 |
| Ilex cornuta | Senecio chrysanthemoides | 2 | 0 |
| Ilex cornuta | Herba Patriniae | 2 | 0 |
| Ilex cornuta | Flos Inulae | 2 | 0 |
| Ilex cornuta | Camptotheca acuminata | 2 | 0 |
| Ilex cornuta | Xanthoceras sorbifolia | 2 | 0 |
| Ilex cornuta | Homo sapiens | 2 | 0 |
| Ilex cornuta | Artemisia apiacea | 2 | 0 |
| Ilex cornuta | Periostracum Cicadae | 2 | 0 |
| Ilex cornuta | Reineckea carnea | 2 | 0 |
| Ilex cornuta | Pteris cretica var. nervosa | 2 | 0 |
| Ilex cornuta | Cuscuta chinensis | 2 | 0 |
| Ilex cornuta | Herba Acalyphae | 2 | 0 |
| Ilex cornuta | Pyrola calliantha | 2 | 0 |
| Ilex cornuta | Ziziphus jujuba | 2 | 0 |
| Ilex cornuta | Artemisia capillaris | 2 | 0 |
| Ilex cornuta | Sedum lineare | 2 | 0 |
| Ilex cornuta | Folium Pini | 2 | 0 |
| Ilex cornuta | Piper kadsura | 2 | 0 |
| Ilex cornuta | Fructus Mori | 2 | 0 |
| Ilex cornuta | Typha angustata | 2 | 0 |
| Ilex cornuta | Radix Paeoniae Rubra | 2 | 0 |
| Ilex cornuta | Tagetes patula | 2 | 0 |
| Ilex cornuta | Loranthus parasiticus | 2 | 0 |
| Ilex cornuta | Rheum tanguticum | 2 | 0 |
| Ilex cornuta | Thuja orientalis | 2 | 0 |
| Ilex cornuta | Fagopyrum tataricum | 2 | 0 |
| Ilex cornuta | Rheum officinale | 2 | 0 |
| Ilex cornuta | Citrus medica var. sarcodactylis | 2 | 0 |
| Ilex cornuta | Caulis Clematidis Armandii | 2 | 0 |
| Ilex cornuta | Rhododendron dauricum | 2 | 0 |
| Ilex cornuta | Fructus Ligustri lucidi | 2 | 0 |
| Ilex cornuta | Echinacea purpurea | 2 | 0 |
| Ilex cornuta | Apocynum lancifolium | 2 | 0 |
| Ilex cornuta | Thalictrum ichangense | 2 | 0 |
| Ilex cornuta | Fructus Caryophylli | 2 | 0 |
| Ilex cornuta | Hypericum japonicum | 2 | 0 |
| Ilex cornuta | Panax pseudo - ginseng var. notoginseng | 2 | 0 |
| Ilex cornuta | Pygeum topengii | 2 | 0 |
| Ilex cornuta | Equisetum hiemale | 2 | 0 |
| Ilex cornuta | Pericarpium Arecae | 2 | 0 |
| Ilex cornuta | Rhodiola crenulata | 2 | 0 |
| Ilex cornuta | Fructus Evodiae | 2 | 0 |
| Ilex cornuta | Rosa chinensis | 2 | 0 |
| Ilex cornuta | Arctium lappa | 2 | 0 |
| Ilex cornuta | Ginkgo biloba | 2 | 0 |
| Ilex cornuta | Rhododendron mucronulatum | 2 | 0 |
| Ilex cornuta | Syzygium samarangense | 2 | 0 |
| Ilex cornuta | Scolopendra subspinipes mutilans | 2 | 0 |
| Ilex cornuta | Caesalpinia sappan | 2 | 0 |
| Ilex cornuta | Trifolium pratense | 2 | 0 |
| Ilex cornuta | Artemisia annua | 2 | 0 |
| Ilex cornuta | Tremlla fuciformis | 2 | 0 |
| Ilex cornuta | Sorbaria sorbifolia | 2 | 0 |
| Ilex cornuta | Senecio scandens | 2 | 0 |
| Ilex cornuta | Carthamus tinctorius | 2 | 0 |
| Ilex cornuta | Nidus Vespae | 2 | 0 |
| Ilex cornuta | Eucommia ulmoides | 2 | 0 |
| Ilex cornuta | Rhodomyrtus tomentosa | 2 | 0 |
| Ilex cornuta | Dysosma versipellis | 2 | 0 |
| Ilex cornuta | Uncaria sinensis | 2 | 0 |
| Ilex cornuta | Cynomorium songaricum | 2 | 0 |
| Ilex cornuta | Centella asiatica | 2 | 0 |
| Ilex cornuta | Diaphragma juglandis | 2 | 0 |
| Ilex cornuta | Hippophae rhamnoides | 2 | 0 |
| Ilex cornuta | Solanum nigrum | 2 | 0 |
| Ilex cornuta | Polygonum aviculare | 2 | 0 |
| Ilex cornuta | Stephania cepharantha | 2 | 0 |
| Ilex cornuta | Sophora japonica | 2 | 0 |
| Ilex cornuta | Rubus idaeus | 2 | 0 |
| Ilex cornuta | Rhododendron huianum | 2 | 0 |
| Ilex cornuta | Paeonia albiflora | 2 | 0 |
| Ilex cornuta | Euphorbia humifusa | 2 | 0 |
| Ilex cornuta | Phellodendron amurense | 2 | 0 |
| Ilex cornuta | Sesamum indicum | 2 | 0 |
| Ilex cornuta | Apocynum venetum | 2 | 0 |
| Ilex cornuta | Potentilla chinensis | 2 | 0 |
| Ilex cornuta | Massa Medicata Fermentata | 2 | 0 |
| Ilex cornuta | Rhizoma Typhonii | 2 | 0 |
| Ilex cornuta | Cinnamomum cassia | 2 | 0 |
| Ilex cornuta | Trigonella foenum -graecum | 2 | 0 |
| Ilex cornuta | Houttuynia cordata | 2 | 0 |
| Ilex cornuta | Hypericum perforatum | 2 | 0 |
| Ilex cornuta | pericarpium pyri | 2 | 0 |
| Ilex cornuta | Typha latifolia | 2 | 0 |
| Ilex cornuta | Fructus Schiandrae | 2 | 0 |
| Ilex cornuta | Radix Pseudostellariae | 2 | 0 |
| Ilex cornuta | Cacumen Tamaricis | 2 | 0 |
| Ilex cornuta | Alpinia officinarum | 2 | 0 |
| Ilex cornuta | Asparagus cochinchinensis | 2 | 0 |
| Ilex cornuta | Taxus brevifolia | 2 | 0 |
| Ilex cornuta | Clerodendron cyrtophyllum | 2 | 0 |
| Ilex cornuta | Paederia scandens | 2 | 1 |
| Ilex cornuta | Herba Cirsii Japonici | 2 | 1 |
| Ilex cornuta | Semen Sojae Praeparata | 2 | 1 |
| Ilex cornuta | Radix Cudraniae | 2 | 1 |
| Ilex cornuta | Herba Cichorii | 2 | 1 |
| Ilex cornuta | Taraxacum mongolicum | 2 | 1 |
| Ilex cornuta | Chrysanthemum indicum | 2 | 1 |
| Ilex cornuta | Rheum wittrocki | 2 | 1 |
| Ilex cornuta | Angelica sinensis | 2 | 1 |
| Ilex cornuta | Typhonium giganteum | 2 | 1 |
| Ilex cornuta | Curcuma longa | 2 | 1 |
| Ilex cornuta | Elsholtzia splendens | 2 | 1 |
| Ilex cornuta | Smilax menispermoidea | 2 | 1 |
| Ilex cornuta | Testa Arachidis Hypogaeae | 2 | 1 |
| Ilex cornuta | Semen Astragali Complanati | 2 | 1 |
| Ilex cornuta | Concha Margaritifera Usta | 2 | 1 |
| Ilex cornuta | Ephedra sinica | 2 | 1 |
| Ilex cornuta | Fagopyrum esculentum | 2 | 1 |
| Ilex cornuta | Herba Dracocephali tangutici | 2 | 1 |
| Ilex cornuta | Fraxinus ornus | 2 | 1 |
| Ilex cornuta | Morus alba | 2 | 1 |
| Ilex cornuta | Citrus limon | 2 | 1 |
| Ilex cornuta | Lygodium japonicum | 2 | 1 |
| Ilex cornuta | Achillea millefolium | 2 | 1 |
| Ilex cornuta | Veratrum album | 2 | 1 |
| Ilex cornuta | Smilax glabra | 2 | 1 |
| Ilex cornuta | Veratrum nigrum var. ussuriense | 2 | 1 |
| Ilex cornuta | spirulina | 2 | 1 |
| Ilex cornuta | Artemisia argyi | 2 | 1 |
| Ilex cornuta | Centipeda minima | 2 | 1 |
| Ilex cornuta | Polygonum cuspidatum | 2 | 1 |
| Ilex cornuta | Potentilla discolor | 2 | 1 |
| Ilex cornuta | Silybum marianum | 2 | 1 |
| Ilex cornuta | Pteris multifida | 2 | 1 |
| Ilex cornuta | Abrus precatorius | 2 | 1 |
| Ilex cornuta | Taraxacum officinale | 2 | 1 |
| Ilex cornuta | Fructus Syzygii | 2 | 1 |
| Ilex cornuta | Vatica rassak | 2 | 1 |
| Ilex cornuta | Lamiophlomis rotata | 2 | 1 |
| Ilex cornuta | Polygonum multiflorum | 2 | 1 |
| Ilex cornuta | Apis mellifera ligustica | 2 | 1 |
| Ilex cornuta | Holothuria leucospilota | 2 | 1 |
| Ilex cornuta | herba meconpsis integrifoliae | 2 | 1 |
| Ilex cornuta | Commelina communis | 2 | 1 |
| Ilex cornuta | Saussurea medusa | 2 | 1 |
| Ilex cornuta | Veratrum grandiflorum | 2 | 1 |
| Ilex cornuta | Fructus Choerospondiatis | 2 | 1 |
| Ilex cornuta | Pericarpium Citri Reticulatae | 2 | 1 |
| Ilex cornuta | Coix lacryma- jobi var. ma - yuen | 2 | 1 |
| Ilex cornuta | caulis Rubi idaei | 2 | 1 |
| Ilex cornuta | Acanthus ilicifolius | 2 | 1 |
| Ilex cornuta | Margarita | 2 | 1 |
| Ilex cornuta | Sagittaria sagittifolia | 2 | 1 |
| Ilex cornuta | Vitis vinifera | 2 | 1 |
| Ilex cornuta | Herba Dendrobii | 2 | 1 |
| Ilex cornuta | Semen Phaseoli | 2 | 1 |
| Ilex cornuta | Ootheca Mantidis | 2 | 1 |
| Ilex cornuta | Cornu Cervi Pantotrichum | 2 | 4 |
| Ilex cornuta | Panax ginseng | 2 | 4 |
| Panax ginseng | Sinoadina racemosa | 3 | 7 |
| Panax ginseng | Symplocos chinensis | 3 | 7 |
| Panax ginseng | Rhododendron ovatum | 3 | 7 |
| Panax ginseng | Phyllanthus emblica | 3 | 7 |
| Panax ginseng | Abelmoschus manihot | 3 | 7 |
| Panax ginseng | Typha angustifolia | 3 | 7 |
| Panax ginseng | Pterospermum lanceaefolium | 3 | 7 |
| Panax ginseng | Tamarix chinensis | 3 | 7 |
| Panax ginseng | Inula britannica | 3 | 7 |
| Panax ginseng | Aster tataricus | 3 | 7 |
| Panax ginseng | Herba Artemisiae Scopariae | 3 | 7 |
| Panax ginseng | Acanthopanax senticosus | 3 | 7 |
| Panax ginseng | Pyrrosia lingua | 3 | 7 |
| Panax ginseng | Rhododendron micranthum | 3 | 7 |
| Panax ginseng | Fructus Xanthii sibirici | 3 | 7 |
| Panax ginseng | Senecio chrysanthemoides | 3 | 7 |
| Panax ginseng | Flos Inulae | 3 | 7 |
| Panax ginseng | Xanthoceras sorbifolia | 3 | 7 |
| Panax ginseng | Artemisia apiacea | 3 | 7 |
| Panax ginseng | Reineckea carnea | 3 | 7 |
| Panax ginseng | Pteris cretica var. nervosa | 3 | 7 |
| Panax ginseng | Cuscuta chinensis | 3 | 7 |
| Panax ginseng | Pyrola calliantha | 3 | 7 |
| Panax ginseng | Ziziphus jujuba | 3 | 7 |
| Panax ginseng | Sedum lineare | 3 | 7 |
| Panax ginseng | Tagetes patula | 3 | 7 |
| Panax ginseng | Loranthus parasiticus | 3 | 7 |
| Panax ginseng | Thuja orientalis | 3 | 7 |
| Panax ginseng | Rhododendron dauricum | 3 | 7 |
| Panax ginseng | Fructus Ligustri lucidi | 3 | 7 |
| Panax ginseng | Apocynum lancifolium | 3 | 7 |
| Panax ginseng | Fructus Caryophylli | 3 | 7 |
| Panax ginseng | Hypericum japonicum | 3 | 7 |
| Panax ginseng | Equisetum hiemale | 3 | 7 |
| Panax ginseng | Rhodiola crenulata | 3 | 7 |
| Panax ginseng | Fructus Evodiae | 3 | 7 |
| Panax ginseng | Rhododendron mucronulatum | 3 | 7 |
| Panax ginseng | Syzygium samarangense | 3 | 7 |
| Panax ginseng | Artemisia annua | 3 | 7 |
| Panax ginseng | Sorbaria sorbifolia | 3 | 7 |
| Panax ginseng | Carthamus tinctorius | 3 | 7 |
| Panax ginseng | Rhodomyrtus tomentosa | 3 | 7 |
| Panax ginseng | Uncaria sinensis | 3 | 7 |
| Panax ginseng | Polygonum aviculare | 3 | 7 |
| Panax ginseng | Rhododendron huianum | 3 | 7 |
| Panax ginseng | Euphorbia humifusa | 3 | 7 |
| Panax ginseng | Apocynum venetum | 3 | 7 |
| Panax ginseng | Potentilla chinensis | 3 | 7 |
| Panax ginseng | Massa Medicata Fermentata | 3 | 7 |
| Panax ginseng | Hypericum perforatum | 3 | 7 |
| Panax ginseng | pericarpium pyri | 3 | 7 |
| Panax ginseng | Typha latifolia | 3 | 7 |
| Panax ginseng | Alpinia officinarum | 3 | 7 |
| Panax ginseng | Clerodendron cyrtophyllum | 3 | 7 |
| Panax ginseng | Patrinia villosa | 3 | 8 |
| Panax ginseng | Hericium erinaceus | 3 | 8 |
| Panax ginseng | Agrimonia pilosa var. japonica | 3 | 8 |
| Panax ginseng | Herba Patriniae | 3 | 8 |
| Panax ginseng | Herba Acalyphae | 3 | 8 |
| Panax ginseng | Artemisia capillaris | 3 | 8 |
| Panax ginseng | Folium Pini | 3 | 8 |
| Panax ginseng | Fructus Mori | 3 | 8 |
| Panax ginseng | Typha angustata | 3 | 8 |
| Panax ginseng | Radix Paeoniae Rubra | 3 | 8 |
| Panax ginseng | Citrus medica var. sarcodactylis | 3 | 8 |
| Panax ginseng | Caulis Clematidis Armandii | 3 | 8 |
| Panax ginseng | Pygeum topengii | 3 | 8 |
| Panax ginseng | Arctium lappa | 3 | 8 |
| Panax ginseng | Caesalpinia sappan | 3 | 8 |
| Panax ginseng | Trifolium pratense | 3 | 8 |
| Panax ginseng | Nidus Vespae | 3 | 8 |
| Panax ginseng | Centella asiatica | 3 | 8 |
| Panax ginseng | Diaphragma juglandis | 3 | 8 |
| Panax ginseng | Hippophae rhamnoides | 3 | 8 |
| Panax ginseng | Stephania cepharantha | 3 | 8 |
| Panax ginseng | Sophora japonica | 3 | 8 |
| Panax ginseng | Paeonia albiflora | 3 | 8 |
| Panax ginseng | Phellodendron amurense | 3 | 8 |
| Panax ginseng | Sesamum indicum | 3 | 8 |
| Panax ginseng | Rhizoma Typhonii | 3 | 8 |
| Panax ginseng | Cinnamomum cassia | 3 | 8 |
| Panax ginseng | Houttuynia cordata | 3 | 8 |
| Panax ginseng | Radix Pseudostellariae | 3 | 8 |
| Panax ginseng | Cacumen Tamaricis | 3 | 8 |
| Panax ginseng | Fructus Kochiae | 3 | 9 |
| Panax ginseng | Amomum villosum | 3 | 9 |
| Panax ginseng | Thalictrum ichangense | 3 | 9 |
| Panax ginseng | Panax pseudo - ginseng var. notoginseng | 3 | 9 |
| Panax ginseng | Senecio scandens | 3 | 9 |
| Panax ginseng | Dysosma versipellis | 3 | 9 |
| Panax ginseng | Rubus idaeus | 3 | 9 |
| Panax ginseng | Fructus Schiandrae | 3 | 9 |
| Panax ginseng | Chrysanthemum indicum | 3 | 10 |
| Panax ginseng | Elsholtzia splendens | 3 | 10 |
| Panax ginseng | Fraxinus ornus | 3 | 10 |
| Panax ginseng | Centipeda minima | 3 | 10 |
| Panax ginseng | Echinacea purpurea | 3 | 10 |
| Panax ginseng | Ginkgo biloba | 3 | 10 |
| Panax ginseng | Apis mellifera ligustica | 3 | 10 |
| Panax ginseng | herba meconpsis integrifoliae | 3 | 10 |
| Panax ginseng | Saussurea medusa | 3 | 10 |
| Panax ginseng | Acanthus ilicifolius | 3 | 10 |
| Panax ginseng | Asparagus cochinchinensis | 3 | 10 |
| Panax ginseng | Astragalus membranaceus | 3 | 11 |
| Panax ginseng | Colla Corii Asini | 3 | 11 |
| Panax ginseng | Rhizoma Fagopyri Cymosi | 3 | 11 |
| Panax ginseng | Isatis indigotica | 3 | 11 |
| Panax ginseng | Juncus effusus | 3 | 11 |
| Panax ginseng | Achillea millefolium | 3 | 11 |
| Panax ginseng | Piper kadsura | 3 | 11 |
| Panax ginseng | Eucommia ulmoides | 3 | 11 |
| Panax ginseng | Commelina communis | 3 | 11 |
| Panax ginseng | Bupleurum chinense | 3 | 12 |
| Panax ginseng | Herba Cichorii | 3 | 12 |
| Panax ginseng | Radix Hedysari | 3 | 12 |
| Panax ginseng | Mentha haplocalyx | 3 | 12 |
| Panax ginseng | Scrophularia ningpoensis | 3 | 12 |
| Panax ginseng | Herba Sedi sarmentosi | 3 | 12 |
| Panax ginseng | Potentilla discolor | 3 | 12 |
| Panax ginseng | Pteris multifida | 3 | 12 |
| Panax ginseng | Lamiophlomis rotata | 3 | 12 |
| Panax ginseng | Scolopendra subspinipes mutilans | 3 | 12 |
| Panax ginseng | Semen Juglandis | 3 | 13 |
| Panax ginseng | Rheum palmatum | 3 | 13 |
| Panax ginseng | Chaenomeles sinensis | 3 | 13 |
| Panax ginseng | Homo sapiens | 3 | 13 |
| Panax ginseng | Rheum tanguticum | 3 | 13 |
| Panax ginseng | Silybum marianum | 3 | 13 |
| Panax ginseng | Taraxacum officinale | 3 | 13 |
| Panax ginseng | Solanum nigrum | 3 | 13 |
| Panax ginseng | Taxus brevifolia | 3 | 13 |
| Panax ginseng | Rheum officinale | 3 | 14 |
| Panax ginseng | Abrus precatorius | 3 | 14 |
| Panax ginseng | Trigonella foenum -graecum | 3 | 14 |
| Panax ginseng | Dioscorea hypoglauca | 3 | 15 |
| Panax ginseng | Semen Sojae Praeparata | 3 | 16 |
| Panax ginseng | Rheum wittrocki | 3 | 18 |
| Panax ginseng | Smilax menispermoidea | 3 | 18 |
| Panax ginseng | Testa Arachidis Hypogaeae | 3 | 18 |
| Panax ginseng | Herba Dracocephali tangutici | 3 | 18 |
| Panax ginseng | Morus alba | 3 | 18 |
| Panax ginseng | Veratrum album | 3 | 18 |
| Panax ginseng | Veratrum nigrum var. ussuriense | 3 | 18 |
| Panax ginseng | Vatica rassak | 3 | 18 |
| Panax ginseng | Polygonum multiflorum | 3 | 18 |
| Panax ginseng | Veratrum grandiflorum | 3 | 18 |
| Panax ginseng | Vitis vinifera | 3 | 18 |
| Panax ginseng | Semen Phaseoli | 3 | 18 |
| Panax ginseng | Radix Cudraniae | 3 | 20 |
| Panax ginseng | Polygonum cuspidatum | 3 | 21 |
| Panax ginseng | Smilax glabra | 3 | 22 |
| Panax ginseng | Tremlla fuciformis | 3 | 23 |
| Panax ginseng | Curcuma longa | 3 | 24 |
| Panax ginseng | Rosa chinensis | 3 | 24 |
| Panax ginseng | Herba Dendrobii | 3 | 24 |
| Panax ginseng | Taraxacum mongolicum | 3 | 25 |
| Panax ginseng | Gardenia jasminoides | 3 | 25 |
| Panax ginseng | Periostracum Cicadae | 3 | 25 |
| Panax ginseng | Concha Margaritifera Usta;Margarita | 3 | 26 |
| Panax ginseng | caulis Rubi idaei | 3 | 26 |
| Panax ginseng | Margarita | 3 | 26 |
| Panax ginseng | Angelica sinensis | 3 | 28 |
| Panax ginseng | Typhonium giganteum | 3 | 29 |
| Panax ginseng | Herba Cirsii Japonici | 3 | 30 |
| Panax ginseng | Citrus limon | 3 | 30 |
| Panax ginseng | Pericarpium Citri Reticulatae | 3 | 30 |
| Panax ginseng | Ootheca Mantidis | 3 | 30 |
| Panax ginseng | Sagittaria sagittifolia | 3 | 32 |
| Panax ginseng | Semen Astragali Complanati | 3 | 33 |
| Panax ginseng | spirulina | 3 | 33 |
| Panax ginseng | Holothuria leucospilota | 3 | 33 |
| Panax ginseng | Cistanche deserticola | 3 | 37 |
| Panax ginseng | Cornu Cervi Pantotrichum | 3 | 67 |
